# Single‐trial log transformation is optimal in frequency analysis of resting EEG alpha

**DOI:** 10.1111/ejn.13854

**Published:** 2018-02-19

**Authors:** Fren T. Y. Smulders, Sanne ten Oever, Franc C. L. Donkers, Conny W. E. M. Quaedflieg, Vincent van de Ven

**Affiliations:** ^1^ Department of Cognitive Neuroscience Faculty of Psychology and Neuroscience Maastricht University Maastricht The Netherlands; ^2^ Department of Clinical Psychological Science Faculty of Psychology and Neuroscience Maastricht University Maastricht The Netherlands

**Keywords:** alpha peak frequency, alpha‐scaling, EEG alpha frequency, transformation

## Abstract

The appropriate definition and scaling of the magnitude of electroencephalogram (EEG) oscillations is an underdeveloped area. The aim of this study was to optimize the analysis of resting EEG alpha magnitude, focusing on alpha peak frequency and nonlinear transformation of alpha power. A family of nonlinear transforms, Box–Cox transforms, were applied to find the transform that (a) maximized a non‐disputed effect: the increase in alpha magnitude when the eyes are closed (Berger effect), and (b) made the distribution of alpha magnitude closest to normal across epochs within each participant, or across participants. The transformations were performed either at the single epoch level or at the epoch‐average level. Alpha peak frequency showed large individual differences, yet good correspondence between various ways to estimate it in 2 min of eyes‐closed and 2 min of eyes‐open resting EEG data. Both alpha magnitude and the Berger effect were larger for individual alpha than for a generic (8–12 Hz) alpha band. The log‐transform on single epochs (a) maximized the *t*‐value of the contrast between the eyes‐open and eyes‐closed conditions when tested within each participant, and (b) rendered near‐normally distributed alpha power across epochs and participants, thereby making further transformation of epoch averages superfluous. The results suggest that the log‐normal distribution is a fundamental property of variations in alpha power across time in the order of seconds. Moreover, effects on alpha power appear to be multiplicative rather than additive. These findings support the use of the log‐transform on single epochs to achieve appropriate scaling of alpha magnitude.

## Introduction

The electroencephalogram (EEG) in the absence of stimulus events, or ‘resting EEG’, is typically characterized by oscillations. One main interest concerns the magnitude of these oscillations or, more specifically, differences between experimental conditions in magnitude. The interpretation of these differences and the statistical power to detect them are likely to be influenced by the manner of quantification and scaling of this magnitude. We focus on the magnitude of alpha (e.g., 8–13 Hz) activity because alpha activity often dominates the EEG, and has been originally associated with cortical de‐arousal or ‘idling’ (e.g., Pfurtscheller, 1992; Pfurtscheller *et al*., 1996), and more recently with inhibition (e.g., Mazaheri & Jensen, 2010), perception, attention, and memory processes (e.g., Thut *et al*., 2006; Klimesch *et al*., 2007; Palva & Palva, 2007; Jensen *et al*., 2014). Furthermore, alpha is strongly increased in an eyes‐closed as compared to an eyes‐open condition, one of the earliest and probably least disputed observations on the human EEG (Berger, 1929). Exactly because the effect of eye closure is so well established, we can use it to ‘tune’ EEG quantification and scaling until our analysis shows maximal sensitivity to this effect (Roberts, 2008). This approach is taken here.

Scaling is important in the definition of alpha magnitude because the results and interpretation of statistical tests depend on it. The magnitude of EEG oscillations does not have a natural scale, contrary to a variable like behavioral response time where the scale can be argued to be linear, thus allowing a strong interpretation of additive effects (Townsend, 1992). For example, if the magnitude of alpha serves as an index of cortical activation, we cannot know *a priori* which scaled version of it shows a preferred linear relation to the underlying construct. Commonly used analyses for hypothesis testing, such as analysis of variance (anova), *t*‐tests, and Pearson's correlation, are insensitive to linear transformation of the data: It does not matter whether potentials are expressed in units of millivolts or microvolts, or whether a constant is added to all measurements. Frequency analysis, however, is usually performed using Fourier analysis, which is typically programmed to yield amplitude, power, or the log of power. These units are easily converted to each other using simple transformations such as square root, square, log, and power transforms, but these transformations are not linear. Therefore, many common statistical tests of alpha magnitude can – and do – show different results, depending on the chosen transformation. Thus, investigators are forced to make a choice among the various options for scaling, and as it is a choice that matters, it should be a deliberate choice.

In most authoritative handbooks and guidelines papers (e.g., Pivik *et al*., 1993; Davidson *et al*., 2000; Pizzagalli, 2007; Keil *et al*., 2014), only a few sources are cited (Davidson *et al*., 1990, footnote 2; Gasser *et al*., 1982) when making recommendations for the transformation of EEG in frequency analysis. These sources evaluated either only the square root and log‐transform (Davidson *et al*., 1990, in 21 undergraduate students), or a somewhat larger set of transforms (Gasser *et al*., 1982, in 31 children). We evaluated 28 transforms, all of them were members of the Box–Cox ‘family’ of power transforms (Box & Cox, 1964) that (a) includes most transforms evaluated earlier, and (b) extends both the range and precision of determining the optimal transformation in a larger (*N* = 87) student population. For defining the ‘optimal’ transformation, we took into account both normality and statistical power of various types of tests. As a comparison, the sometimes used relative spectrum was also added to the set of transforms.

To select the transform that is ‘optimal’, that is, among its competitors it is the most sensitive to an effect that is known to exist and thus has highest statistical power, we need a criterion for sensitivity. Here, we limited ourselves to the *t*‐test because it is simple, powerful, and often used. Furthermore, anova can be regarded as a generalization of the *t*‐test, as it will produce results that will be either identical to *t*‐test results for the simple (i.e., two‐level comparison) designs that we use, or sensitive to transformation in a similar way. We excluded nonparametric tests because they tend to have reduced power, and are generally insensitive to monotonic transformation of the data. But even for a simple *t*‐test, we can distinguish various broad types of analysis in which statistical tests for differences between experimental conditions are meaningful. The first are participant‐level analyses, used when all conditions are presented to each participant a number of times, in different ‘trials’ or ‘epochs’, and a test for the difference is carried out for each participant, using ‘epochs’ as the source of error variance. To establish generality of the effect, the test may be repeated for multiple participants. The second are group‐level analyses, when first a summary measure is computed for each of the participants, that each engage in multiple experimental conditions, creating a ‘within‐design’. Subsequent statistical testing is then carried out using ‘participants’ as the source of error variance. For each of these two types of analysis, the distribution of the data entering the analysis may be quite different. Therefore, the ‘best’ transform may, or may not, be the same for every type.

An additional factor that affects results is the precise definition of alpha frequency. Although alpha is often defined generically as the band from 8–12 or 8–13 Hz (Pizzagalli, 2007), it has been recognized that individuals differ in their dominant alpha frequency (Doppelmayr *et al*., 1998; Klimesch, 1999; Bazanova & Vernon, 2014; Haegens *et al*., 2014; Quaedflieg *et al*., 2015). We aimed to gather additional support for the concept of individual alpha frequency (IAF) by close examination of individual EEG spectra. After that, the effect of transformations was investigated for both generic alpha frequency (GAF) and IAF. First, the effects of various transformations were investigated for the participant‐level analyses (hereafter named level 1 analyses). Then, the effect of the same transformations was investigated for group‐level within‐analyses (level 2 analyses).

## Materials and methods

### Participants

Participants were recruited from a population of students that were enrolled in an EEG practical course of their Master program at Maastricht University. From this population (*n *=* *372, mean age = 23.7 years, SD = 3.0, 56% female), 99 participants volunteered and gave consent to participate after they were informed. Due to technical failure (5), low recording quality resulting in excessive noise (5), or non‐compliance with task instructions (2), the data of 12 participants were excluded from further analysis. The study was approved by the Ethics Review Committee of the Faculty of Psychology and Neuroscience.

### Procedure

Participants were comfortably seated in a dimly lit, sound attenuated room at a distance of about 57 cm from a monitor connected to a stimulus computer. After an electrode cap was placed and good signal quality was verified, participants were invited to produce various types of artifacts. During this period, signal feedback was given to the participant to increase awareness of the sensitivity of EEG measurements. After this, the participants were asked to relax and follow the instructions on the monitor. First, an ‘eyes‐open’ condition was presented, during which the participant only had to watch a fixation dot for 2 min. Then, participants were instructed to close their eyes, and the ‘eyes‐closed’ condition started and was maintained for 2 min. Participant compliance with these instructions was verified using a closed‐circuit television camera. After this resting EEG phase, various short tasks were presented that are not relevant for the current report.

### Data recording and analysis

Signals were recorded from 13 sintered Ag/AgCl electrodes (Fz, Cz, Pz, Oz, F3, F4, C3, C4, P3, P4, O1, O2, A2) positioned in an elastic cap (Easycap) according to the International 10–20 system using a BrainAmp amplifier and BrainVision Recorder software (BrainProducts, Germany). An electrode at the left mastoid bone (A1) served as the recording reference, and an electrode at AFz as ground. Electrode pairs above and below the right eye and at the outer canthi of both eyes recorded vertical and horizontal EOG, respectively. Electrode impedance was kept below 5 kOhm. Signals were sampled at 250 Hz, with a low pass filter set to 70 Hz, a notch filter at 50 Hz, and a time constant of 10 s. The stimulus computer sent trigger signals every 1000 ms to a second computer that also recorded the EEG. These triggers specified each condition as ‘eyes‐open’ or ‘eyes‐closed’.

Off‐line analysis started with rereferencing to the average of A1 and A2 (‘computer‐linked mastoids’), and computing bipolar vertical (VEOG) and horizontal (HEOG) EOG. Eye‐blink artifacts were corrected using a regression procedure based on Semlitsch *et al*. (1986). In each condition, 120 epochs of 512 sample points (2048 ms) were cut, starting at every trigger, thus showing approximately 50% overlap.

Electroencephalogram epochs with extreme voltages (range exceeding 150 μV) were excluded. If more than ten (of 120) epochs were excluded in either the eyes‐open or eyes‐closed condition, data were visually inspected. Based on that, in five cases, it was decided that data quality was too low for further analysis; in one case, normal EEG exceeded 150 μV, and the range criterion was set to 200 μV. In the resulting datasets (*N *=* *87), the average number of epochs per participant was 119.2 with eyes open, and 118.7 with eyes closed. The minima were 105 and 102, respectively.

Pre‐processing for the Fourier transform consisted of (a) subtracting the time‐mean and linear trend of each epoch from the voltage at each time point; (b) windowing, using a Hann window extending over 100% of the epoch; (c) padding with zero's to 1024 points per epoch. Then followed the Fourier transform on the epochs of 4096 ms, resulting in estimates of power in μV^2^ at frequencies separated by 0.244 Hz, the frequency resolution. Computations were performed using EEGLAB version v12.0.2.6b and custom‐written software in Matlab (MathWorks, Inc) version R2014a.

### Alpha frequency and alpha band

#### Generic alpha peak frequency

Generic alpha peak frequency was chosen at 10.01 Hz, the estimate that was closest to 10 Hz.

#### Individual alpha peak frequency

Individual alpha peak frequency was estimated in four steps. In step 1, the epoch‐average power was computed for each frequency and participant in the eyes‐closed condition. In step 2, for improved stability, the spectrum was smoothed with a simple 2‐iteration 5‐point moving average filter. In step 3, the IAF was determined as the frequency between 5 and 15 Hz that had the highest local maximum (‘dominant’) in the epoch‐average power spectrum at the Pz electrode (Klimesch *et al*., 1993). In the rare cases when there was no local maximum because magnitudes were monotonously increasing or decreasing, the algorithm selected 10.01 Hz as IAF, the most conservative choice among arbitrary alternatives for comparability to the GAF. Being based on the eyes‐closed condition, we will denote this estimate as ‘IAF‐ec’. Various alternatives for this ‘peak frequency’ method have been proposed, but they are less often used or lack appealing simplicity (Goljahani *et al*., 2012), or are only required for EEG during a task other than resting (Klimesch *et al*., 1999). Below, it is shown that, in resting EEG data, the simple peak frequency method gave good results in a large sample of participants.

Furthermore, to obtain support for a functional interpretation of IAF, we again determined these estimates in the eyes‐open condition and in the difference between spectra between eyes‐closed and eyes‐open, as computed just before step 2. This added, respectively, IAF‐eo and IAF‐eceo to our analysis.

#### Generic and individual alpha band

In view of the results of alpha peak frequency analyses, presented below, we selected both the generic and the individual alpha peak frequency that was based on the eyes‐closed condition (IAF‐ec) as the basis to define the alpha frequency band. For both individual and generic alpha frequency (AF), the alpha band was bounded by AF ± 0.20 × AF or the closest frequency that was available, given the resolution of 0.244 Hz (Doppelmayr *et al*., 1998). The resulting single‐epoch‐based estimates of alpha band power formed one set of data that were entered into the transformations.

#### Alpha magnitude: transformations

Transformations were performed on alpha power at the Pz electrode according to the Box–Cox procedure (Box & Cox, 1964; Hoaglin *et al*., 1983; Roberts, 2008). In this approach, the transforms are of the form:

(1) $$x^{\prime }=\left\{\begin{array}{c} \frac{x^{p}-1}{p},\;\;p\neq 0\\ \log_{e}x,\;\;p=0 \end{array}\right.$$

**Figure 1:**
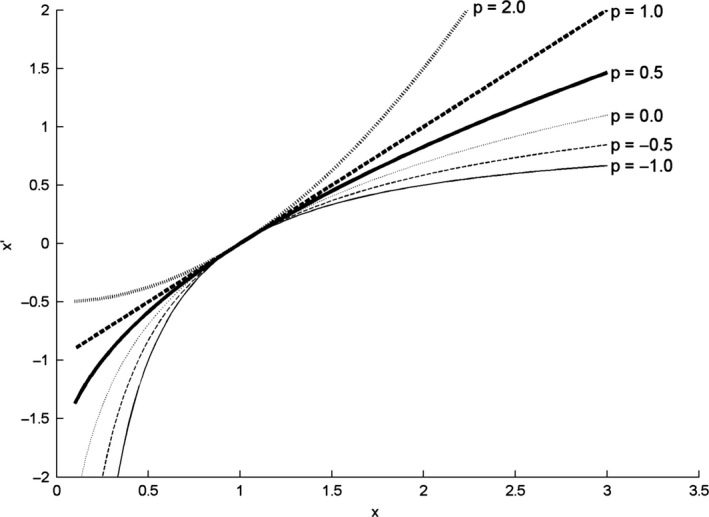
Power functions (Eqn (1)), for selected values of *p*.

For a detailed description of this family of transforms, see Hoaglin *et al*. (1983, p. 98–104). A few features are noteworthy here. First, Fig. 1 shows *x*′ as a function of *x* for various values of −1 ≤ *p *≤* *2. It can be seen that as *p* increases, the function gradually changes from strongly concave down (*p *<* *1) to straight (*p *=* *1) to concave up (*p *>* *1). As a result, lower values of *p* make up transforms that are capable of transforming more positively skewed distributions to normal. Secondly, Fig. 1 suggests correctly that the log‐transform (*p *=* *0) fits between *p *=* *−0.5 and *p *=* *0.5. *p*‐Values even closer to zero yield transforms that approach the log‐transform even more. Therefore, the log‐transform is a natural member of the family rather than an odd one out. Thirdly, except for *p *=* *0, the transforms are linear transforms of power functions *x* ^*p*^. Statistics that are insensitive to linear rescaling (*t*‐tests, anova, etc.) therefore yield the same results for the Box–Cox transforms as for simple power functions with the same *p*. In fact, the only reason to prefer Eqn (1) over *x* ^*p*^ is that all curves in Fig. 1 are increasing and go through a single point (1,0). Fourth, and related to the previous remark, many transforms that have been used for EEG power in the past are equivalent to members of the Box–Cox family: square root (*p *=* *0.5), 1/*x* (*p *=* *−1), cubic root (*p *=* *1/3), untransformed power (*p *=* *1), and the log‐transform (*p *=* *0). Fifth, the base of the log does not matter to the results of *t*‐tests or anovas, as log‐transforms with a different base are linearly related, as shown by the change‐of‐base formula: log_*a*_(*x*) = log_*b*_(*x*)/log_*b*_(*a*). We used *e *≈* *2.7183 as base, rendering the natural log (ln). Finally, applying one member of the family to amplitude is mathematically equivalent to applying another one to power, because (*a* ^*b*^)^*p*^ = *a* ^b*p^, so provided that an appropriate series of values of *p* is tested, there is no need to do the transforms on both amplitude (where *b* = 0.5) and power, separately.

Likely, the *p*‐value that yields the ‘best’ results depends upon the distribution of alpha magnitude across trials.

We applied the transformations for a range of 28 *p*‐values (−1.5 to 1.2 in steps of 0.1) and evaluated how the results depended on them for two statistics. First, we contrasted the eyes‐open and the eyes‐closed conditions with a *t*‐test. Larger *t*‐values suggested a higher statistical power, given the assumption that the difference between the conditions is real. Second, for both the eyes‐open and eyes‐closed conditions, we evaluated the normality of the distribution of the transformed data, using D'Agostino's statistic *K* ^2^ which takes into account both symmetry and kurtosis (Zar, 1999; Trujillo‐Ortiz & Hernandez‐Walls, 2003). It was possible that the transform that yielded the highest statistical power to find a known experimental effect was also the transform that generated a distribution of alpha that was closest to normal.

Importantly, we performed the transformations both at the level of the single epochs, that is, preceding participant‐level analyses (level 1), and at the level of the epoch averages, preceding group‐level analyses (level 2). This allowed comparison of the two analysis scenarios. At the single‐epoch level, the transforms used were:

(2) $${E_{i,j}}^{\prime }=\left\{\begin{array}{c} \frac{{E_{i,j}}^{p1}-1}{p1},\;\;p1\neq 0\\ \log_{e}E_{i,j},\;\;p1=0 \end{array}\right.$$

with E_*i,j*_ being alpha power in the alpha band in epoch *i*, participant *j*, and *p1* being the power value used for transforms at the single‐epoch level. At the epoch‐average level, we first computed for each participant

(3) $$A_{j}=\frac{\sum_{i=1}^{n_{j}}E_{i,j}}{n_{j}}$$

with A_*j*_ being the alpha band magnitude in participant *j*, averaged across *n*_*j*_ epochs, and then transformed the data as:

(4) $${A_{j}}^{\prime }=\left\{\begin{array}{c} \frac{{A_{j}}^{p2}-1}{p2},\;\;p2\neq 0\\ \log_{e}A_{j},\;\;p2=0 \end{array}\right.$$

with *p2* being the *p*‐value used for transforms at the participant level (level 2).

Note that a combination of transforms at both the single epoch and epoch‐average level meets with the difficulty that the result of Eqn (2) can be non‐positive, while the input of any Box–Cox transform must be positive. Therefore, only the epoch average of untransformed alpha power was used as input in Eqn (4).

Finally, for comparison, the optimal transform within the Box–Cox family was compared to the relative spectrum, defined as the ratio between the epoch‐average power (untransformed) within the alpha band and the summed power across all frequencies (1–30 Hz).

## Results

### Generic and individual alpha peak frequency

**Figure 2:**
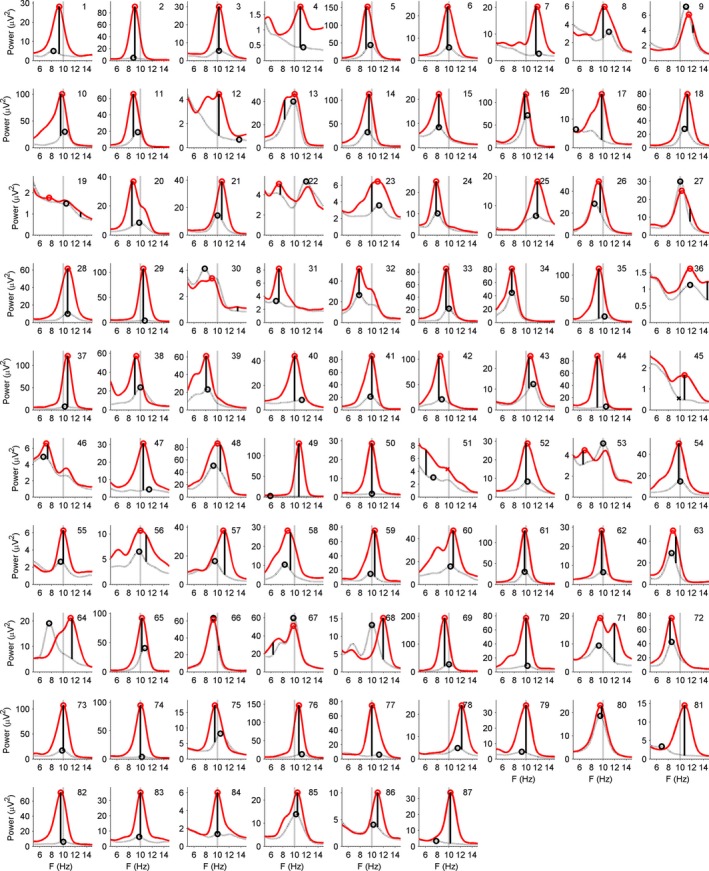
Power spectra, averaged across epochs, as a function of EEG frequency in the eyes‐closed (solid) and eyes‐open (dotted) conditions for each individual participant (1, 2, …, 87). Peak frequencies have been marked with an open circle. In case the spectrum displayed no local peak, an *X* was plotted at 10.01 Hz. A vertical solid line connecting the ‘eyes closed’ and the ‘eyes open’ line marks the frequency that shows the largest positive difference between them. The vertical dotted line marks the generic alpha frequency of 10.01 Hz.

Figure 2 shows power spectra in the frequency range 5–15 Hz for every individual participant in the eyes‐closed and eyes‐open conditions at the Pz electrode. Some observations can be made. First, inspection of the scaling of the *y*‐axis demonstrates that there was a large variation among participants in alpha power. For example, across participants 3, 4, and 5, the power spanned about two orders of magnitude. Yet, remarkably, despite this large variation, there existed a clearly dominant frequency in the alpha range, at least in the eyes‐closed condition. A direct consequence of this large variation is that the participants with a low magnitude have only a weak effect on the shape, and peak frequency, of the participant‐average spectrum. Further, those participants in the group for which the effect is large dominate the frequency at which the experimental effect is highest at the group level. Importantly, outlier rejection was neither straightforward nor desirable as the pattern of results of these participants did not meaningfully deviate from the rest of the sample. Instead, some form of normalization or transformation could provide a solution to this issue, which we discuss in detail, below.

**Figure 3:**
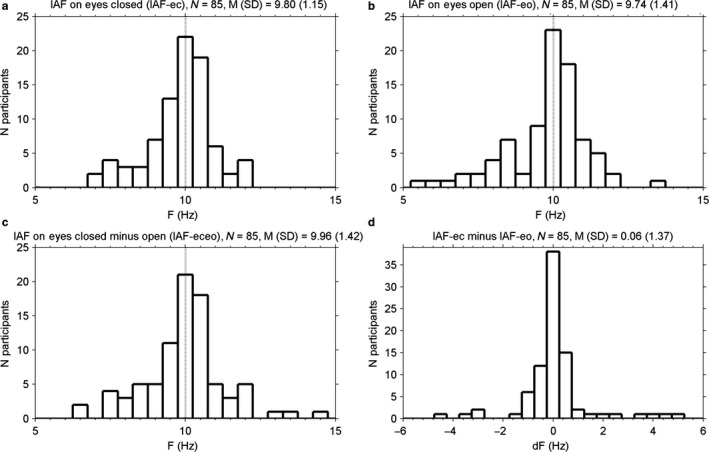
Histograms of individual alpha frequency (IAF), as obtained in (a) the power spectrum of the eyes‐closed condition, (b) the power spectrum of the eyes‐open condition, and (c) the difference in power between the eyes‐closed and eyes‐open conditions. (d). A histogram of the difference between IAFs obtained in eyes‐closed and eyes‐open conditions (IAF‐ec minus IAF‐eo). F = Frequency; dF = difference in frequency.

Second, with eyes‐closed, the large majority of participants showed a single, clear peak in this frequency range, unambiguously defining their IAF‐ec. Peak frequencies have been marked by an open circle symbol in the plots. With eyes closed, in one participant (no. 51, marked with an asterisk), there was no peak. In the eyes‐open condition, there was also no peak in one case (no. 45), although power values were generally smaller, and the selected peak frequency seemed more arbitrary in some participants (4, 7, 12, 17, 31, 37, 44, 47, 49, 50, 51, 77, 82, 84, 87). The two cases without a peak have been omitted from the histograms in Fig. 3 and further IAF analyses, so that *n *=* *85 remained. For the estimation of alpha magnitude, IAF was replaced by the GAF in these cases, as detailed in the Methods.

Third, clearly, there was substantial variation in the frequency at which alpha showed a peak, so that it often deviated from GAF, which is marked with the vertical dotted line in Fig. 2. In Fig. 3, panel (a) shows a histogram of IAF‐ec. IAF‐ec differed more than 0.50 Hz from GAF in 46 of 85 participants (54%). The distribution of IAF‐ec across participants closely resembled the one reported by Bazanova & Vernon (2014, their Fig. 2) in terms of range, central value, and shape.

Fourth, in Fig. 2, the vertical solid line connecting the eyes‐open and eyes‐closed curves marks the frequency at which the difference in power was maximal (IAF‐eceo). Figure 3, panel (c) shows the associated distribution across participants. In many cases, IAF‐eceo was very close to IAF‐ec. The difference between IAF‐eceo and IAF‐ec was smaller than 0.5 Hz in 74 of 85 participants (87%). A *t*‐test showed no significant difference in means (*t*_84_ = 1.44, *P *=* *0.15), and an *F*‐test showed a slightly larger variance in IAF‐eceo (*s* ^2^ = 2.03) than in IAF‐ec (*s* ^2^ = 1.30), *F*_84,84_ = 1.55, *P *=* *0.047. IAF‐eo was within 0.5 Hz of IAF‐eceo in 39 of 85 participants (46%), and neither means (*t*_84_ = 1.21, *P *=* *0.23) nor variances (*F*_84,84_ = 1.02, *P *=* *0.93) differed significantly.

Fifth, in 54 of 85 participants (64%), IAF‐ec differed less than 0.50 Hz from IAF‐eo. In some of the cases where the difference seemed especially large, the power values with eyes‐open were low and lacked a large peak, rendering the outcome of peak picking unreliable (see point 2, above). In some other cases (no. 57, 64, 68), though, there were large peaks in both the eyes‐open and eyes‐closed conditions, and there was a large difference between IAF‐ec and IAF‐eo. Interestingly, in those cases, IAF‐ec was higher than IAF‐eo. However, this counterintuitive observation concerned only a small subsample of our participants and was not supported by a statistical test on IAF‐ec vs. IAF‐eo on the whole sample (IAF‐ec: M (SD) = 9.80 (1.15), IAF‐eo: M (SD) = 9.74 (1.41), *t*_84_ = 0.41, *P *=* *0.69). In Fig. 3, panel (b) shows a histogram of IAF‐eo, and panel (d) shows a histogram of IAF‐ec minus IAF‐eo.

Sixth, the histogram of IAF‐ec in Fig. 3, panel (a), shows that IAF‐ec, even while being allowed to vary within a wide range of 5–15 Hz, was distributed symmetrically around GAF of 10.01 Hz (the dotted vertical line). It is also obvious that a generic alpha band (e.g., 8–12 Hz) in most of the participants would capture the peak that defined IAF‐ec. Still, as the IAF peak falls closer to a border of the generic alpha band, that band does not capture an increasing proportion of the power in the individual alpha band, leading to an underestimation of alpha power.

In sum, the results suggest that (a) IAF can reliably be found especially in the eyes‐closed condition, (b) it deviates clearly from GAF in many participants, and (c) it shows a bell‐shaped distribution among participants. Moreover, IAF in the eyes‐closed condition is almost always close to the frequency that changes the most when the eyes are open vs. closed, and often also close to the IAF in the eyes‐open condition. This can be taken as supporting evidence for a functional interpretation of individual alpha, and it is especially encouraging in light of the known threats to validity associated with peak picking, which have been documented in detail for event‐related potentials by Luck (2014), and, in principle, could hold for frequency spectra as well. In the remainder of the text, whenever ‘IAF’ is used, it refers to IAF as obtained in the eyes‐closed condition, unless explicitly stated otherwise. For comparison, GAF was further investigated as well and its sensitivity was compared to IAF after the optimal transformation was selected, as reported below.

### Transformations on single epochs

Below, we use *t1* to refer to (results from) participant‐level *t*‐tests that were performed for each individual participant (level 1), and *t2* to denote (results from) group‐level *t*‐tests (level 2). The effects of transformations on the generic alpha band were highly similar to effects on the individual alpha band, unless stated otherwise, so we mainly discuss the latter. Detailed results for the generic alpha band can be obtained in the Supporting Information.

**Figure 4:**
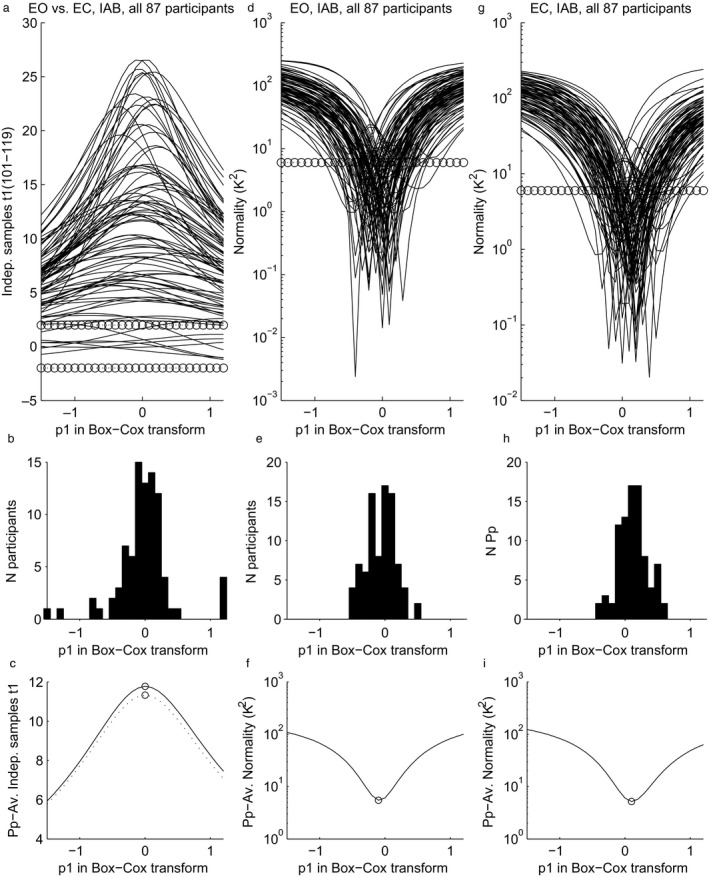
Effects of *p1* in transformations of power in the individual alpha band at the single‐epoch level (Eqn (2)) on analyses at participant level (level 1, see text). (a) Statistical power (*t*1‐value) of the contrast between eyes‐open and eyes‐closed, for all participants. (b) Histogram of the peaks of the waveforms depicted in panel a. (c) Solid line: average across participants of the waveforms depicted in panel a, so for the individual alpha band. Dotted line: the same, but now for the generic alpha band (not plotted in panel a and b). (d) Normality (*K* ^2^) of the distribution across epochs in the eyes‐open condition, for all participants. Lower values indicate a closer approximation to normal. (e) Histogram of the troughs depicted in panel d. (f) Average across participants of the waveforms depicted in panel d. (g) Normality (*K* ^2^) of the distribution across epochs in the eyes‐closed condition, for all participants (lower is more normal). (h) Histogram of the troughs depicted in panel g. (i) Average across participants of the waveforms depicted in panel g. The number of non‐artefactual epochs varied among participants; therefore, degrees of freedom for the *t*‐tests ranged from 101 to 119. Circles mark critical *t*1 values for *Df* = 100 at alpha = 0.05 (two‐sided) in panel (a), and critical *K* ^2^ values at alpha = 0.05 (two‐sided) in panels (d) and (g).

In Fig. 4, panel (a) shows the effect of parameter *p1* in the Box–Cox transform (Eqn (2)) on the *t1*‐value associated with the independent‐samples *t*‐test that compares, within each participant, alpha magnitude in the individual alpha band in eyes‐open and eyes‐closed epochs. First, it can be seen that the range of tested *p1* values was sufficiently wide: for nearly all participants, there was a peak indicating the highest (optimal) *t1* value within the tested range, and closer to the edges of the tested range of *p1* values, curves were generally decreasing.

Second, Fig. 4, panel (b) shows a histogram of the *N *=* *87 values of *p1* that were peaks in panel (a), that is, that were optimal for a participant. The large majority of peaks were in the range −0.5 to +0.5, with most of them clustered around *p1 *=* *0, corresponding to the log‐transform. In Fig. 4, panel (c) shows the participant‐average of the waveforms in panel (a). For *p1 *=* *0, it attained its highest value, showing that if a single value of *p1* must be selected for all participants, then the optimal choice is the log‐transform. Although the statistical power at *p1 *=* *1 (equivalent to ‘no transform’) was certainly not low (eyes closed vs. eyes open is a strong effect), there was a substantial gain in power at *p1 *=* *0. On average, across participants, comparing the *t1*'s of *p1 *=* *0 and *p1 *=* *1 yielded means of 11.8 and 8.3, respectively. A group‐level *t2*‐test showed a significant difference (*t2*_86_ = 9.91, *P *<* *0.0005).

In Fig. 4, panel (d) shows the effect of *p1* on normality of the distribution of alpha magnitude across epochs in the eyes‐open condition, with lower values indicating a closer approximation to normal. The histogram in panel (e) shows that for every participant, the closest approximation was obtained with a value of *p1* ranging between −0.5 and 0.5 and the optimal *p1* values were symmetrically clustering around *p1 *=* *0, the log‐transform. Panel (f) shows the participant‐average of the waveforms in panel (d). When *p1 *=* *−0.1, it attained its lowest value, showing that if a single value of *p1* is to be used for all participants, then the *p1 *=* *−0.1, yields, on average, the most normal distribution. This value of *p1 *=* *−0.1 was again very close to the log‐transform (*p1 *=* *0). Panels (g, h, i) show the same relations as panels (d, e, f), but now for the eyes‐closed condition, with similar results. Only this time the average in panel (i) was minimal for *p1 *=* *+0.1, so still close to zero. Although participant‐mean optimal *p1* was close to zero in both the eyes‐open and eyes‐closed conditions, a group‐level *t2*‐test nevertheless revealed a significant difference (eyes‐open: M = −0.062, eyes‐closed: M = 0.121, *t2*_86_ = 5.95, *P *<* *0.0005).

### Comparing transformations on epoch averages and single epochs

While significance testing at the participant level (level 1) is affected by the distribution across epochs, the group‐level testing (level 2) is affected by the distribution across participants. The latter distribution can be modified by transformations either at the single‐epoch level (Eqn (2), level 1, as in the previous section) or at the epoch‐average level (Eqn (4), level 2). When transforming epoch averages (Eqn (4)), we used the untransformed epoch‐average power (Eqn (3)) as input. This averaging of untransformed power values at level 1 is recommended by Oppenheim & Schafer (2010) and seems to be the default used by most EEG investigators (e.g., Tomarken *et al*., 1992; Keil *et al*., 2014). Thus, the comparison can be described as ‘level 1‐only’ transform vs. a ‘level 2‐only’ transform.

**Figure 5:**
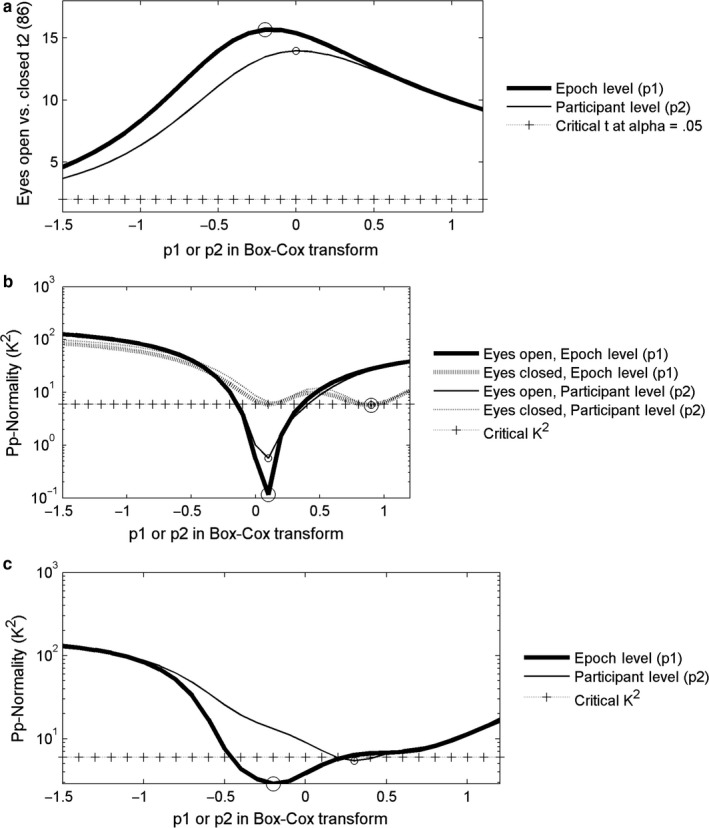
Effects of *p1* and *p2* in power transformations of power in the individual alpha band at the single epoch level (*p1*, Eqn (2)) and epoch‐average level (*p2*, Eqn (4)) on analyses at the group level (level 2, see text). (a) Statistical power (*t2*‐value) of the contrast between eyes‐open and eyes‐closed. (b). Normality (*K* ^2^) of the distribution across participants in the eyes‐open and eyes‐closed conditions. (c). Normality (*K* ^2^) of the distribution across participants of the difference between eyes‐closed and eyes‐open conditions.

In Fig. 5, panel (a) shows the effects of these transformations on the *t2*‐value of the contrast between alpha magnitude in eyes‐open and eyes‐closed conditions. First, the level‐2 transforms yielded a maximal *t2*‐value for *p2 *=* *0, corresponding to the log‐transform. Second, the level 1 transforms yielded a maximal *t2*‐value for *p1 *=* *−0.2. Third, even at *p1 *=* *0, the *t2* is still larger for epoch‐level transformation than with *p2 *=* *0 for participant‐level transformation. The results for generic alpha power were highly similar and can be obtained in the supplementary online information.

In Fig. 5, panel (b) shows the effect of *p1* and *p2* on normality of the distribution across participants (*K* ^2^) in eyes‐open and eyes‐closed conditions. In the eyes‐closed condition, normality was most closely approached at *p1* or p*2 *=* *0.9, and at *p1* or *p2* of 0.1 in the eyes‐open condition. Only in the latter case, there was an advantage for the epoch‐level transformation over the participant‐level transformation.

More relevant to paired‐comparison *t*‐testing, however, is the normality of the distribution of differences between eyes‐closed and eyes‐open conditions. This normality is depicted in panel (c). For participant‐level transformations, the optimal *p2* was 0.3, and for epoch‐level transformation, the optimal *p1* was −0.2 and lower values of *K* ^2^ were attained.

### Individual vs. generic alpha band

The above findings indicate that epoch‐level transformations that are equal or close to the log‐transform are optimal. Therefore, this log‐transform was selected for further statistical comparison of the generic and individual alpha band. Two tests were performed. The *t1*‐values of independent‐sample *t*‐tests contrasting eyes‐open and eyes‐closed in each individual participant were on average larger for the IAB than for the GAB (level‐2 paired‐comparison *t*‐test: IAB: M (SD) = 11.8 (7.2), GAB: M = 11.3 (7.3), *t2*_86_ = 4.0, *P *<* *0.0005). Also, an anova with state (eyes‐open, eyes‐closed) and band (IAB, GAB) as factors showed alpha magnitude was larger with eyes closed than with eyes open (*F*_1,86_ = 223.7, *P *<* *0.0005), and larger for the IAB than for the GAB (*F*_1,86_ = 9.4, *P *=* *0.003). Furthermore, the difference between the eyes‐open and eyes‐closed states was larger for the IAB than with the GAB (*F*_1,88_ = 17.0, *P *<* *0.0005). These effects support the hypothesis that the individually defined alpha band has greater sensitivity than the generic alpha band.

### Single‐epoch log‐transform vs. relative power

The single‐epoch log‐transform was also selected for comparison to alpha magnitude taken from the relative power spectrum. Relative power is commonly tested at the group level, so we did the same, and compared to the single‐epoch log‐transform (*p1 *=* *0) of absolute power. The paired‐comparison *t2*‐value associated with the difference between the eyes‐open and eyes‐closed conditions was higher for the single‐epoch log‐transformed absolute alpha power than for the relative alpha power (*t2*_86_ = 15.38, *P *<* *0.0005 and *t2*_86_ = 11.38, *P *<* *0.0005, respectively). Furthermore, normality of the distribution of the eyes‐closed and eyes‐open difference contrast across participants was rejected for relative alpha (D’ Agostino *K* ^2^ = 10.16, *P *=* *0.006), but not for the single‐epoch log‐transform (*K* ^2^ = 3.84, *P *=* *0.147).

### SD as a function of mean

**Figure 6:**
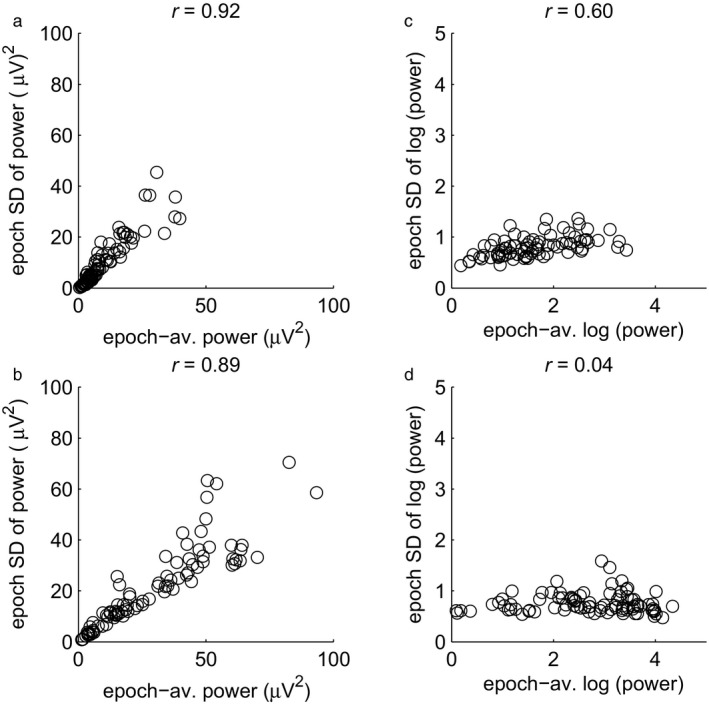
The relation between the average and SD of alpha magnitude across epochs in the individual alpha band. One dot corresponds to one participant. (a) Epoch SD of untransformed alpha power as a function of epoch‐average untransformed alpha power in the eyes‐open condition. (b) The same, in the eyes‐closed condition. (c) Epoch SD of the log of alpha power as a function of the epoch‐average log‐transformed alpha power in the eyes‐open condition. (d) The same, in the eyes‐closed condition.

The above results suggested that the across‐epoch distribution of EEG alpha power approached normality after taking the log transform on individual epochs. Whenever the distribution of a log‐transformed variable is normal, the distribution of the original, untransformed variable is called ‘log‐normal’. A log‐normal distribution across epochs results if the variable is the product of a large number of (latent) independent, identically distributed variables in the same way that a normal distribution results if the variable is the *sum* of a large number of independent, identically distributed variables (central limit theorem; Weisstein, 2017). Further support for the notion that alpha power in each single epoch is the product of latent variables can be obtained by showing that modulations of alpha power are multiplicative in nature. One type of modulation concerns individual differences. If they are multiplicative, then the epoch standard deviation should increase linearly with the epoch average (Zar, 1999). Figure 6, panels (a) and (b) display the relation between the epoch average and epoch standard deviation in the eyes‐open and eyes‐closed conditions, respectively, a single dot representing one participant. It can be seen that the standard deviation indeed tended to increase linearly with the mean, with linear correlations of *r *=* *0.92 and *r *=* *0.89 for eyes‐open and eyes‐closed, respectively. For log‐transformed data, in panel (c) and (d), the relation was either weaker (eyes‐open: *r *=* *0.60) or absent (eyes closed: *r *=* *0.04). These results suggest that individual differences within conditions are also multiplicative rather than additive.

### Characterizing variation at the original scale of the data

Given the indications that the variation in alpha power among epochs follows a log‐normal distribution, and that the single‐epoch log‐transform is optimal, this transform would be preferred for statistical testing. However, one disadvantage of the log‐transform is that the results in terms of absolute numeric values are less familiar than at the original scale (μV^2^). Thus, for plotting, one would like to characterize the epoch‐average data on their original scale. Traditionally, the mean and standard deviation of log *x* would be used to characterize the data, but they may be ‘back‐transformed’ to yield the multiplicative (or geometric) mean $\overset{¯}{x}*$ and multiplicative standard deviation s* (Limpert *et al*., 2001; Limpert & Stahel, 2011). These parameters have some notable features. First, they determine an interval containing 68.3% of the data in the same way as the description $\overset{¯}{x}\pm \;\text{SD}$ does for (additive) normal data. The interval ranges from $\overset{¯}{x}*$ divided by s* to $\overset{¯}{x}*$ times s* and can be denoted as $\overset{¯}{x}*$ *x*/s* (read as ‘$\overset{¯}{x}*$ times‐divide s*’). For example, with $\overset{¯}{x}*$ = 100, and s* = 2, the lower and upper boundaries would be 100/2 = 50 and 100 × 2 = 200, respectively. Note that if the boundaries of the interval would be plotted as error bars on a linear scale, they would be asymmetric, as appropriate for a skewed distribution; but if plotted on a log scale, they are symmetric again. Second, s* characterizes the log‐normal distribution in that the more it deviates from 1, the larger the deviation from normality. Third, s* is scale‐free in the sense that it is not sensitive to the units of the variable (e.g., volt vs. microvolt).

**Figure 7:**
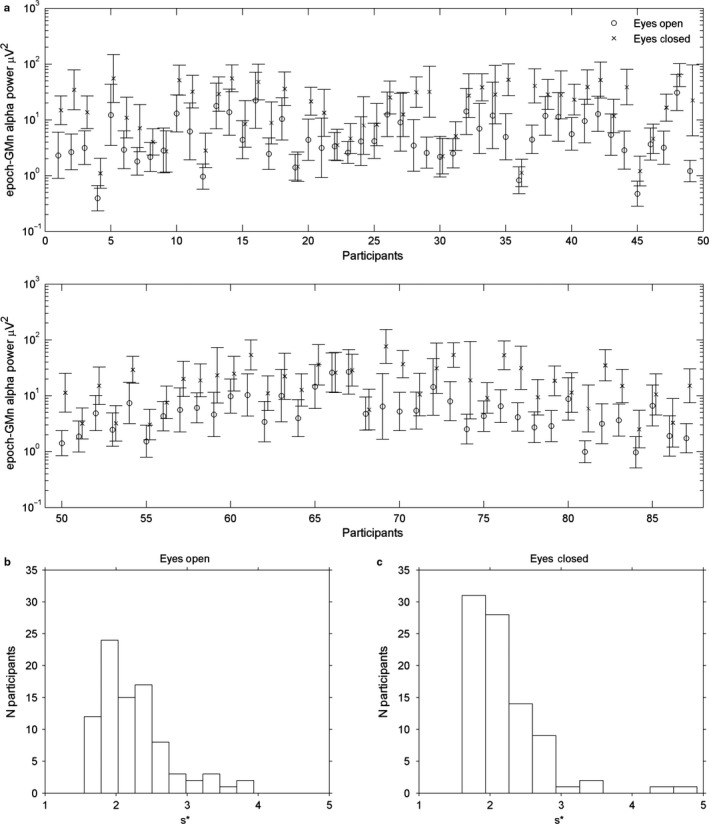
(a) Geometric mean alpha power in the individual alpha band for all 87 participants, plotted on a log scale. Error bars indicate the multiplicative standard deviation s* (see text). For clarity, the data for the eyes‐closed condition have been slightly moved to the right. (b) The distribution of the multiplicative standard deviation s* among participants in the eyes‐open condition. (c) The same as (b), but in the eyes‐closed condition.

In Fig. 7, panel (a) shows geometric epoch‐mean individual alpha power at the Pz electrode in eyes‐open and eyes‐closed conditions, for each participant with error bars indicating s*. Note that in this graph, the same vertical distance indicates the same proportional increase. The vertical distance between eyes‐open and eyes‐closed seems not to depend heavily on the position on the scale, suggesting that the effect of closing the eyes is proportional, or multiplicative, rather than additive. The same holds for the error bars. The plot would look just the same if the epoch‐average (arithmetic mean) of log‐transformed power were plotted on a linear scale. The latter, however, does not offer the advantage of an easily interpretable *y*‐axis. For plotting and interpretation of group data, one can use either the participant‐arithmetic mean of epoch‐geometric means, plotted on a log scale, or the participant‐arithmetic mean of epoch‐log transformed power, plotted on a linear scale.

In Fig. 7, panel (b) and (c) show histograms of s* in eyes‐open and eyes‐closed conditions, respectively. In no participant, s* was smaller than 1.5, and the great majority was between 1.5 and 3. The participant‐mean of s* did not differ significantly between eyes‐open (mean s* = 2.20) and eyes‐closed conditions (mean s* = 2.15) (*t*_88_ = 0.71, *P *=* *0.48).

## Discussion

The premise was that appropriate definition and scaling of the magnitude of EEG oscillations in the alpha frequency range is an underdeveloped area. The aim was to optimize the analysis of resting EEG alpha magnitude, focusing on alpha peak frequency and nonlinear data transformation. The results, however, also have several theoretical implications.

The results supported the concept of IAF. Next to substantial individual variation in alpha magnitude, individual spectra showed peaks in the alpha range at frequencies that were clearly different for individual participants (Fig. 2). When individual alpha peak frequency was estimated in the eyes‐closed condition, where alpha was largest, and where estimates were plausibly most reliable, it was identical to, or close to the frequency that showed the largest difference between eyes‐open and eyes‐closed conditions, and often also close to the peak frequency in the eyes‐open condition. At least some of the deviations from the latter pattern seemed to result from unreliable peak picking in the eyes‐closed–eyes‐open difference, or in the eyes‐open condition, when power or differences in power were small. In sum, individual spectra suggested not only that individual alpha peak frequency is rather stable across conditions that differ substantially in magnitude, but also that it is the frequency that is most sensitive to an experimental manipulation that allegedly changes cortical activation, namely open vs. closed eyes. This adds support to a functional interpretation of IAF, as opposed to being merely a result of random errors in the estimation of a peak in the spectrum. This functional interpretation agrees well with earlier findings that individual differences in alpha frequency are related to interindividual differences in age and several cognitive functions including attention and memory (e.g., Klimesch *et al*., 1993; Klimesch, 1999), and perception (Samaha & Postle, 2015). Across participants, we performed direct statistical tests of the difference between generic and individual alpha power (both epoch‐log transformed). Not only overall alpha magnitude, but also the differences between eyes‐open and eyes‐closed were larger for individual alpha than for generic alpha. The latter result held for both average *t* values of level‐1 *t*‐tests and alpha magnitude.

A family of transformations (Fig. 1) provided a graded correction for right‐skewness of the distribution of alpha power across epochs (level 1) or across participants (level 2). The effect of level‐1 transformation was studied for *t*‐tests and normality of the distribution within each participant. At the group level, we compared level‐1 and level‐2 transformations (Eqn (2) and (4) resp.).

For *t*‐tests and normality within each participant, the log‐transform – or a transform close to it – was optimal (Fig. 4). It yielded, on average, the highest *t*‐value for the eyes‐open vs. eyes‐closed contrast, and it was also the transform that yielded across‐epoch distributions very close to normality. So, if a single transform is to be selected and applied to single epochs in all participants, as is preferable, then the log‐transform is optimal. Furthermore, if a distribution of log‐transformed data is normal, we may call the distribution of the untransformed data ‘log‐normal’, and this has theoretical implications, as outlined below.

Preceding analyses at the group level, there is a choice between two options for transformation: (a) transforming the alpha power in single epochs (level 1), then averaging across epochs, followed by the *t*‐test and evaluation of normality, or (b) first averaging across epochs, transforming the epoch average (level 2), and then do the *t*‐test and evaluation of normality. The highest *t*‐value and smallest deviation from normality were attained when log‐transformation was performed at single‐epoch level. For the paired‐sample *t*‐test at the group level, it is the normality across participants of the difference between eyes‐open and eyes‐closed conditions that matters, rather than the normality of the distribution in each isolated condition. Only level‐1 transformations close to the log‐transform managed to keep the statistic *K* ^2^ far below the threshold for rejection of this normality. Also, the comparison to relative power was favorable for the epoch‐level log‐transform: The relative power yielded a smaller *t*‐value and a significant deviation from normality at the group level for the eyes‐open and eyes‐closed contrast.

Yet another reason to prefer transformation at the single‐epoch level is a more general one. Optimization of measurement (including transformation) within individual participants should be preferred because once a maximally reliable score for each participant has been obtained, only the issues of generalization to the population and possible existence of true individual differences are left for the group‐level analyses.

### Theoretical implications

According to the theorem of central limits, a variable that is the *sum* of a number of independent underlying variables will be normally distributed. If experimental effects concern one of those underlying variables, then anova and *t*‐tests, which test for additive effects on the mean, are appropriate. A variable that is the product of a number of independent underlying variables will be distributed log‐normally (McDonald, 2014), meaning that their log‐transform yields data that are distributed normally and may be tested for additive effects with anova and *t*‐tests [note that the log‐transform turns any multiplicative effects into additive effects, see the basic rule of log computations log(*xy*) = log(*x*) + log(*y*)]. Effects on the untransformed variable will then be multiplicative in nature. One consequence is that effects on the standard deviation will be proportional to the mean (Zar, 1999), as also observed in the present data (Fig. 6).

If it is accepted that the log‐normal distribution is a fundamental property of second‐by‐second variations in EEG‐alpha power across time, it is interesting to speculate about a neural architecture that would generate alpha power as a product of independent random variables. The fact that the variation happens along epochs within single participants rules out many sources of variability such as skull thickness, electrode resistance, and idiosyncratic folding pattern of the cortex. Probably the most simplistic architecture is a series of neural modules acting as ‘amplifiers’, each having an independent randomly variable gain factor. If arranged in a series, the total gain is the product of the individual gains. The start of the series would be the driving generator of alpha, and the end would be pyramidal neurons in the cortex, whose signal is picked up by the EEG electrode.

Our recommendation to average the log‐transformed single‐epoch power values rather than the untransformed power values deviates from the method of averaging periodograms that has been recommended for EEG (Keil *et al*., 2014). The latter recommendation was presumably rooted in the established works of Welch (1967) and Oppenheim & Schafer (2010). It should be taken into account, however, that the latter authors were concerned with the estimation of stationary oscillations in noise. Even brief observation of any raw EEG time series data makes obvious that EEG oscillations are not stationary, but highly variable across time intervals of more than a few seconds. Plausibly, it is the latter variability that necessitates the log‐transform.

Our recommendation to transform the data at the single‐epoch level rather than after epoch‐averaging parallels that by Grandchamp & Delorme (2011). These authors, however, were not concerned with resting EEG, but with event‐related changes in the spectrum with respect to a baseline preceding each individual event. The method that came out as best in their study involved computing *Z*‐scores at single‐epoch level. The involved division by the SD across time might have a similar effect as taking the log, if indeed that SD increases linearly with the mean, as when variations are multiplicative, and log‐normally distributed.

Gasser *et al*. (1982) tried various (level‐2 only) transforms on relative and absolute epoch‐average spectra, and evaluated skewness, kurtosis, and Wilk's *W* of the distribution across participants in various frequency bands. Most relevant to the current discussion are their results about absolute spectra in two alpha bands (7.5–9.5 and 9.5–12.5 Hz). In both cases, the log‐transform performed best in transforming toward normality. Likewise, Davidson *et al*. (1990), comparing the log and square root (level‐2) transform, concluded that the log‐transform resulted in a less‐skewed distribution. As argued above, our data suggest that the same transform on single epochs (level 1) is sufficient to achieve normality and is preferable.

### Limitations

Although we analyzed individual alpha peak frequency in a usual manner, the current results do not tell us anything about the stability of individual differences in the long run, for example, across sessions separated by days or months. Therefore, individual differences might reflect a current ‘state’, or a stable ‘trait’. Still, the results do suggest a degree of stability in alpha peak frequency across conditions (eyes‐open and eyes‐closed) that have a great influence on magnitude. Earlier data from Gasser *et al*. (1985) showed that individual alpha peak frequency is also stable across months. Nevertheless, the peak might still shift during specific tasks, as suggested by Klimesch *et al*. (1993) and Haegens *et al*. (2014). Alpha activity during cognitive tasks is beyond the scope of the present paper.

The current results concern only resting EEG. Although measurement of resting EEG with eyes‐open and eyes‐closed is very common, EEG during other conditions is important in many other studies. We hypothesize that a log‐normal distribution across epochs and multiplicative rather than additive effects characterize EEG in those studies as well, but this needs to be verified. Also, in task‐based paradigms, effects might benefit from transformations not tested here. Based on our results in the eyes‐open condition, suggesting a log‐normal distribution along single epochs even when alpha magnitude is comparatively small (Fig. 4d–f), we would expect beneficial effects especially for transforms that capitalize on proportional rather than additive task effects (see Grandchamp & Delorme, 2011).

## Conclusion and recommendations

Individual alpha peak frequency can be determined reliably in only 2 min in an eyes‐closed condition. This frequency does not differ systematically from an eyes‐open condition, or from the frequency that shows the largest difference in alpha power between eyes‐open and eyes‐closed conditions. Moreover, when it is used to define the individual alpha band, then the latter shows a higher magnitude of alpha and stronger Berger effects than a GAF (8–12 Hz). Thus, we recommend using IAF rather than a single generic alpha frequency.

The current results suggest that the log‐transform on single epochs, so preceding averaging across epochs or trials, is both necessary and sufficient. It is necessary because it converts the log‐normal distribution to normal or near‐normal and transforms multiplicative effects into additive effects. It is sufficient, because it normalizes both the distribution across trials within participants, and the distribution across participants.

In our search for the optimal transformation, we tried many that varied in a graded way. We argue that this was permissible because we studied a known and undisputed effect, the Berger effect. Indeed, we do *not* recommend that many transformations be performed routinely in an attempt to maximize just any experimental effect, as this could be regarded as ‘fishing for significance’. Yet we still encourage replication and generalization of the finding that the log‐transform on single epochs is optimal. That generalization could concern other frequency bands, other states like sleep or after drug intake, or specific participant or patient groups.

## Conflict of interests

The authors declare that there are no conflict of interests.

## Data accessibility

The authors have made the data available: Smulders, Fren T.Y.; Oever, Sanne ten; Donkers, Franc C.L.; Quaedflieg, Conny W.E.M.; Ven, Vincent van de, 2018, ‘Single‐trial log‐transformation is optimal in frequency analysis of resting EEG alpha’, hdl:10411/Z97KYD, DataverseNL Dataverse, V1.

## Author contributions

FS, StO, FD, and CQ collected the data. FS drafted the manuscript. StO, FD, CQ, and VvdV edited the manuscript. VvdV and StO supported data analysis and plotting. All authors approved the final version.

**Abbreviations**
AF: alpha frequency
anova: analysis of variance
ECEO: eyes closed minus eyes open
EC: eyes closed
EEG: electroencephalogram
EO: eyes open
GAB: generic alpha band
GAF: generic alpha frequency
IAB: individual alpha band
IAF: individual alpha frequency

## Supporting information

Fig. S1. Effects of *p1* in transformations of power in the generic alpha band at the single‐epoch level (Eq. 2) on analyses at participant level (level 1, see text).

Fig. S2. Effects of *p1* and *p2* in power transformations of power in the generic alpha band at the single epoch level (*p1*, Eq. 2) and epoch‐average level (*p2*, Eq. 4) on analyses at the group level (level 2, see text).

Fig. S3. The relation between the average and SD of alpha magnitude across epochs in de generic alpha band.

Fig. S4. Geometric mean alpha power in the generic alpha band for all 87 participants, plotted on a log scale.

## References

[ejn13854-bib-0001] Bazanova, O.M. & Vernon, D. (2014) Interpreting EEG alpha activity. Neurosci. Biobehav. R., 44, 94–110. 10.1016/j.neubiorev.2013.05.007 23701947

[ejn13854-bib-0002] Berger, H. (1929) Über das elektrenkephalogramm des menschen. Eur. Arch. Psy. Clin. N., 87, 527–570.

[ejn13854-bib-0003] Box, G.E. & Cox, D.R. (1964) An analysis of transformations. J. Roy. Stat. Soc. B. Met., 26, 211–252.

[ejn13854-bib-0004] Davidson, R.J. , Chapman, J.P. , Chapman, L.J. & Henriques, J.B. (1990) Asymmetrical brain electrical activity discriminates between psychometrically‐matched verbal and spatial cognitive tasks. Psychophysiology, 27, 528–543. 2274616 10.1111/j.1469-8986.1990.tb01970.x

[ejn13854-bib-0005] Davidson, R.J. , Jackson, D.C. & Larson, C.L. (2000) Human electroencephalography In Cacioppo J.T., Tassinary L.G. & Berntson G.G. (Eds), Handbook of Psychophysiology. Cambridge University Press, Cambridge, pp. 27–52.

[ejn13854-bib-0006] Doppelmayr, M. , Klimesch, W. , Pachinger, T. & Ripper, B. (1998) Individual differences in brain dynamics: important implications for the calculation of event‐related band power. Biol. Cybern., 79, 49–57. 9742677 10.1007/s004220050457

[ejn13854-bib-0007] Gasser, T. , Bächer, P. & Möcks, J. (1982) Transformations towards the normal distribution of broad band spectral parameters of the EEG. Electroen. Clin. Neuro., 53, 119–124. 10.1016/0013-4694(82)90112-2 6173196

[ejn13854-bib-0008] Gasser, T. , Bächer, P. & Steinberg, H. (1985) Test–retest reliability of spectral parameters of the EEG. Electroen. Clin. Neuro., 60, 312–319. 10.1016/0013-4694(85)90005-7 2579798

[ejn13854-bib-0009] Goljahani, A. , D'Avanzo, C. , Schiff, S. , Amodio, P. , Bisiacchi, P. & Sparacino, G. (2012) A novel method for the determination of the EEG individual alpha frequency. Neuroimage, 60, 774–786. 22182767 10.1016/j.neuroimage.2011.12.001

[ejn13854-bib-0010] Grandchamp, R. & Delorme, A. (2011) Single‐trial normalization for event‐related spectral decomposition reduces sensitivity to noisy trials. Front. Psychol., 2, 236. 21994498 10.3389/fpsyg.2011.00236 PMC3183439

[ejn13854-bib-0011] Haegens, S. , Cousijn, H. , Wallis, G. , Harrison, P.J. & Nobre, A.C. (2014) Inter‐ and intra‐individual variability in alpha peak frequency. Neuroimage, 92, 46–55. 24508648 10.1016/j.neuroimage.2014.01.049 PMC4013551

[ejn13854-bib-0012] Hoaglin, D.C. , Mosteller, F. & Tukey, J.W. (1983) Understanding Robust and Exploratory Data Analysis, vol 3. Wiley, New York.

[ejn13854-bib-0013] Jensen, O. , Gips, B. , Bergmann, T.O. & Bonnefond, M. (2014) Temporal coding organized by coupled alpha and gamma oscillations prioritize visual processing. Trends Neurosci., 37, 357–369. 24836381 10.1016/j.tins.2014.04.001

[ejn13854-bib-0014] Keil, A. , Debener, S. , Gratton, G. , Junghofer, M. , Kappenman, E.S. , Luck, S.J. , Luu, P. , Miller, G.A. *et al* (2014) Committee report: publication guidelines and recommendations for studies using electroencephalography and magnetoencephalography. Psychophysiology, 51, 1–21. 24147581 10.1111/psyp.12147

[ejn13854-bib-0015] Klimesch, W. (1999) EEG alpha and theta oscillations reflect cognitive and memory performance: a review and analysis. Brain Res. Rev., 29, 169–195. 10209231 10.1016/s0165-0173(98)00056-3

[ejn13854-bib-0016] Klimesch, W. , Schimke, H. & Pfurtscheller, G. (1993) Alpha frequency, cognitive load and memory performance. Brain Topogr., 5, 241–251. 8507550 10.1007/BF01128991

[ejn13854-bib-0017] Klimesch, W. , Sauseng, P. & Hanslmayr, S. (2007) EEG alpha oscillations: the inhibition–timing hypothesis. Brain Res. Rev., 53, 63–88. 16887192 10.1016/j.brainresrev.2006.06.003

[ejn13854-bib-0018] Limpert, E. & Stahel, W.A. (2011) Problems with using the normal distribution – and ways to improve quality and efficiency of data analysis. PLoS One, 6, e21403. 21779325 10.1371/journal.pone.0021403 PMC3136454

[ejn13854-bib-0019] Limpert, E. , Stahel, W.A. & Abbt, M. (2001) Log‐normal distributions across the sciences: keys and clues. Bioscience, 51, 341–352.

[ejn13854-bib-0020] Luck, S.J. (2014) An introduction to the event‐related potential technique, 2 Edn MIT, Cambridge.

[ejn13854-bib-0021] Mazaheri, A. & Jensen, O. (2010) Rhythmic pulsing: linking ongoing brain activity with evoked responses. Front. Hum. Neurosci., 4, 177. 21060804 10.3389/fnhum.2010.00177 PMC2972683

[ejn13854-bib-0022] McDonald, J.H. (2014) Handbook of Biological Statistics, 3rd Edn Sparky House Publishing, Baltimore, MD Available http://www.biostathandbook.com/transformation.html.

[ejn13854-bib-0023] Oppenheim, A.V. & Schafer, R.W. (2010) Discrete‐time Signal Processing, 3rd Edn. Prentice Hall Press Upper Saddle River, NJ.

[ejn13854-bib-0024] Palva, S. & Palva, J.M. (2007) New vistas for α‐frequency band oscillations. Trends Neurosci., 30, 150–158. 17307258 10.1016/j.tins.2007.02.001

[ejn13854-bib-0025] Pfurtscheller, G. (1992) Event‐related synchronization (ERS): an electrophysiological correlate of cortical areas at rest. Electroen. Clin. Neuro., 83, 62–69. 10.1016/0013-4694(92)90133-3 1376667

[ejn13854-bib-0026] Pfurtscheller, G. , Stancak, A. & Neuper, C. (1996) Event‐related synchronization (ERS) in the alpha band—an electrophysiological correlate of cortical idling: a review. Int. J. Psychophysiol., 24, 39–46. 8978434 10.1016/s0167-8760(96)00066-9

[ejn13854-bib-0027] Pivik, R.T. , Broughton, R.J. , Coppola, R. , Davidson, R.J. , Fox, N. & Nuwer, M.R. (1993) Guidelines for the recording and quantitative analysis of electroencephalographic activity in research contexts. Psychophysiology, 30, 547–558. 8248447 10.1111/j.1469-8986.1993.tb02081.x

[ejn13854-bib-0028] Pizzagalli, D.A. (2007) Electroencephalography and high‐density electrophysiological source localization In Cacioppo J.T., Tassinary L.G. & Berntson G.G. (Eds), Handbook of Psychophysiology, 3 Edn Cambridge University Press, Cambridge, pp. 56–84.

[ejn13854-bib-0029] Quaedflieg, C.W.E.M. , Meyer, T. , Smulders, F.T.Y. & Smeets, T. (2015) The functional role of individual‐alpha based frontal asymmetry in stress responding. Biol. Psychol., 104, 75–81. 25481665 10.1016/j.biopsycho.2014.11.014

[ejn13854-bib-0030] Roberts, S. (2008) Transform your data. Nutrition, 24, 492–494. 18321679 10.1016/j.nut.2008.01.004

[ejn13854-bib-0031] Samaha, J. & Postle, B.R. (2015) The speed of alpha‐band oscillations predicts the temporal resolution of visual perception. Curr. Biol., 25, 2985–2990. 26526370 10.1016/j.cub.2015.10.007 PMC4654641

[ejn13854-bib-0032] Semlitsch, H.V. , Anderer, P. , Schuster, P. & Presslich, O. (1986) A solution for reliable and valid reduction of ocular artifacts, applied to the P300 ERP. Psychophysiology, 23, 695–703. 3823345 10.1111/j.1469-8986.1986.tb00696.x

[ejn13854-bib-0033] Thut, G. , Nietzel, A. , Brandt, S.A. & Pascual‐Leone, A. (2006) Alpha‐band electroencephalographic activity over occipital cortex indexes visuospatial attention bias and predicts visual target detection. J. Neurosci., 26, 9494–9502. 16971533 10.1523/JNEUROSCI.0875-06.2006 PMC6674607

[ejn13854-bib-0034] Tomarken, A.J. , Davidson, R.J. , Wheeler, R.E. & Kinney, L. (1992) Psychometric properties of resting anterior EEG asymmetry: temporal stability and internal consistency. Psychophysiology, 29, 576–592. 1410187 10.1111/j.1469-8986.1992.tb02034.x

[ejn13854-bib-0035] Townsend, J.T. (1992) On the proper scales for reaction time In Geissler H.‐G. & Link S.W. (Eds), Cognition, Information Processing, and Psychophysics: Basic Issues. Scientific Psychology Series. Lawrence Erlbaum Associates, Inc., Hillsdale, NJ, pp. 105–120.

[ejn13854-bib-0036] Trujillo‐Ortiz, A. & Hernandez‐Walls, R. (2003) DagosPtest: D'Agostino‐Pearson's K^2^ test for assessing normality of data using skewness and kurtosis. A MATLAB file. Available http://www.mathworks.com/matlabcentral/fileexchange/3954-dagosptest.

[ejn13854-bib-0037] Weisstein, E.W. (2017) “Log normal distribution.” From MathWorld – A Wolfram Web Resource. Available http://mathworld.wolfram.com/LogNormalDistribution.html.

[ejn13854-bib-0038] Welch, P.D. (1967) The use of fast Fourier transform for the estimation of power spectra: a method based on time averaging over short, modified periodograms. IEEE T. Acoust. Spee., AU‐15, 70–73.

[ejn13854-bib-0039] Zar, J.H. (1999) Biostatistical Analysis, 4th Edn Prentice Hall, Upper Saddle River, NJ.

