# Supplementary material for: Single‐trial log transformation is optimal in frequency analysis of resting EEG alpha
Source: Eur J Neurosci. 2018 Feb 19;48(7):2585–98. doi: 10.1111/ejn.13854 (PMC6221126; doi:10.1111/ejn.13854)
Supplement: Supplementary file 2 [file EJN-48-2585-s002.docx]

Supplementary information with Single-trial log transformation is optimal in frequency analysis of resting EEG alpha

Fren T.Y. Smulders, Sanne ten Oever, Franc C.L. Donkers, Conny W.E.M. Quaedflieg, Vincent van de Ven

Figure S1, S2, S3, S4 below are related to Figure 4, 5, 6, 7 in the main paper, respectively. The Figures in the main paper show effects on the *individual alpha band*, and Figure S1,…S4 show effects on the *generic alpha band (GAB)*.

*
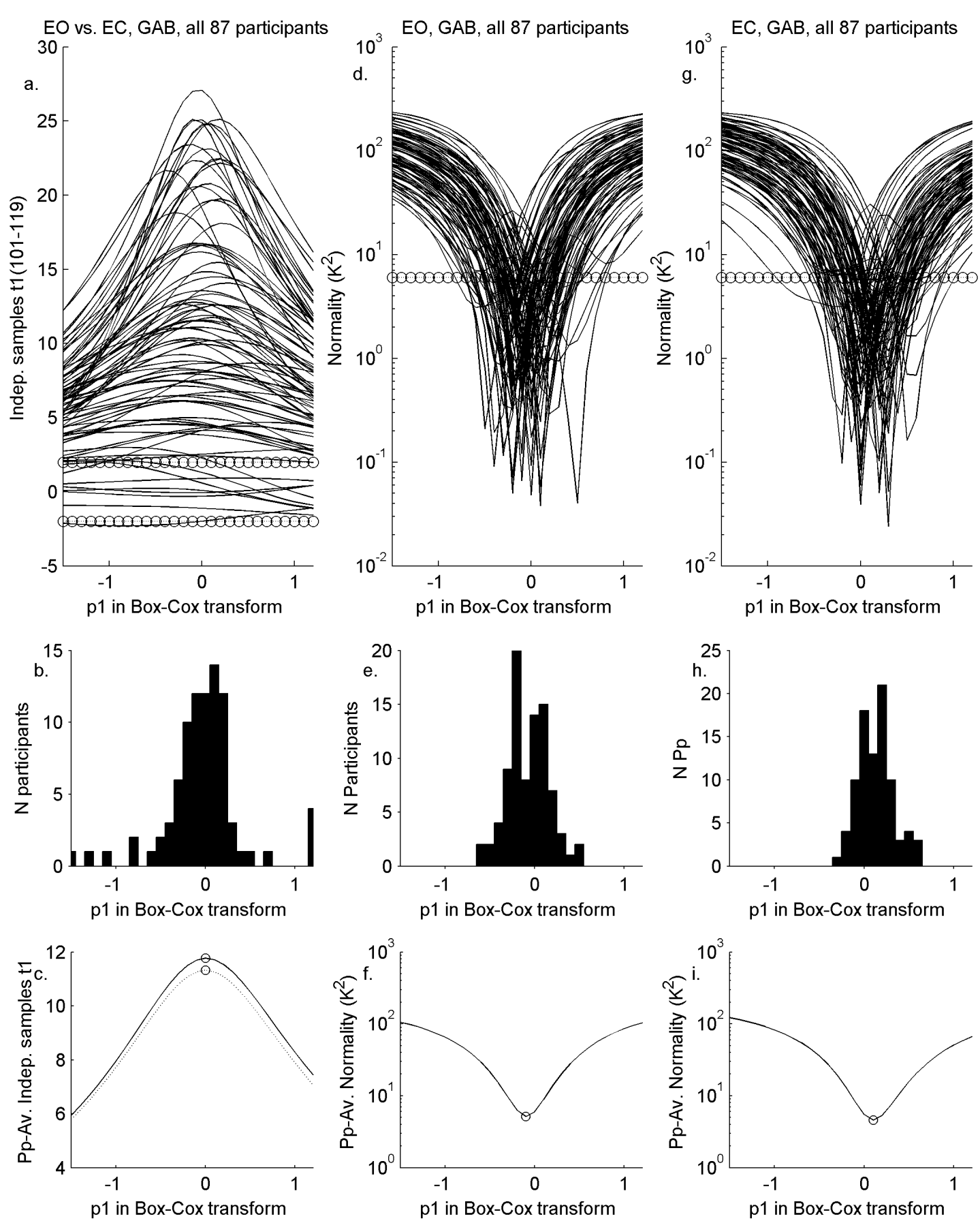
*

*Figure S1.* Effects of *p1* in transformations of power in the generic alpha band at the single-epoch level (Eq. 2) on analyses at participant level (level 1, see text).

a. Statistical power (*t1*-value) of the contrast between eyes-open and eyes-closed, for all participants.

b. Histogram of the peaks of the waveforms depicted in panel a.

c. Dotted line: average across participants of the waveforms depicted in panel a, so for the generic alpha band. Solid line: the same, but now for the individual alpha band (not plotted in panel a and b).

d. Normality (*K*^2^) of the distribution across epochs in the eyes-open condition, for all participants. Lower values indicate a closer approximation to normal.

e. Histogram of the troughs depicted in panel d.

f. Average across participants of the waveforms depicted in panel d.

g. Normality (*K*^2^) of the distribution across epochs in the eyes-closed condition, for all participants (lower is more normal).

h. Histogram of the troughs depicted in panel g.

i. Average across participants of the waveforms depicted in panel g.

The number of non-artefactual epochs varied among participants, therefore degrees of freedom for the *t*-tests ranged from 101 to 119. Circles mark critical *t1*-values for *Df* = 100 at alpha = 0.05 (2-sided) in panel (a), and critical *K*^2^ values at alpha = 0.05 (2-sided) in panel (d) and (g).

*
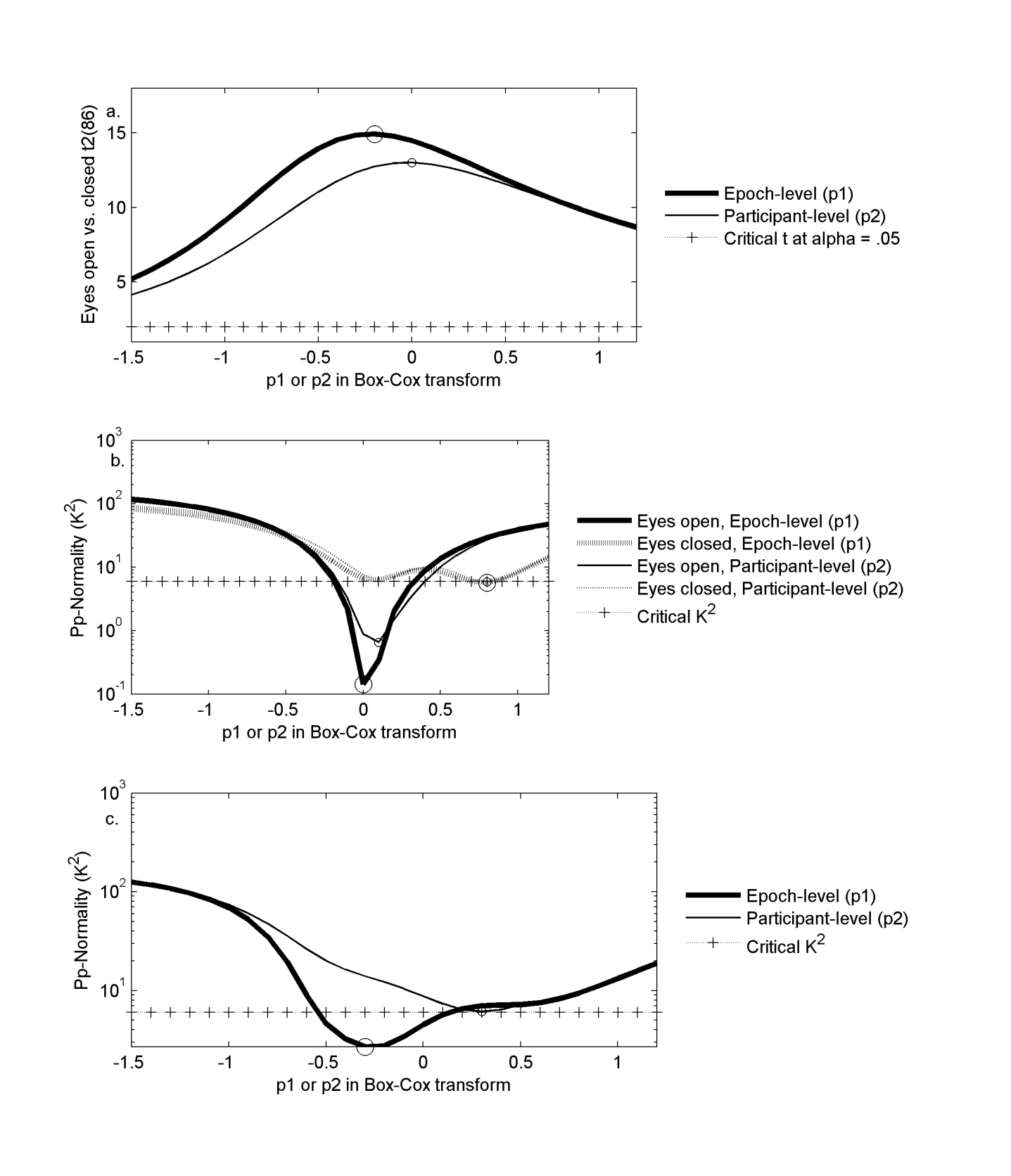
*

*Figure S2.* Effects of *p1* and *p2* in power transformations of power in the generic alpha band at the single epoch level (*p1*, Eq. 2) and epoch-average level (*p2*, Eq. 4) on analyses at the group level (level 2, see text).

a. Statistical power (*t2*-value) of the contrast between eyes-open and eyes-closed.

b. Normality (*K*^2^) of the distribution across participants in the eyes-open and eyes-closed condition.

c. Normality (*K*^2^) of the distribution across participants of the difference between eyes-closed and eyes-open conditions.

*
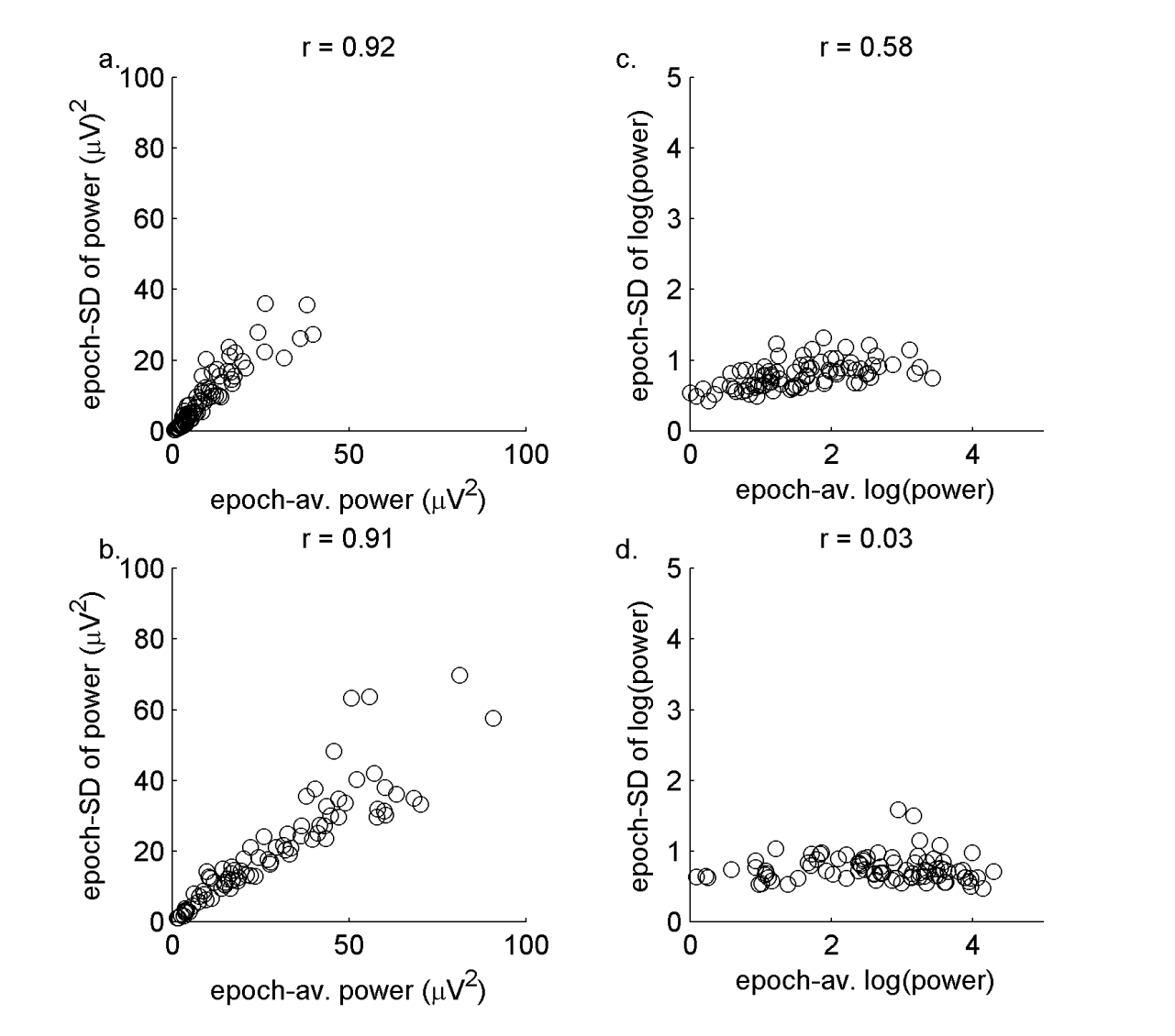
*

*Figure S3*.The relation between the average and SD of alpha magnitude across epochs in de generic alpha band. One dot corresponds to one participant. a. epoch-SD of untransformed alpha power as a function of epoch-average untransformed alpha power in the eyes-open condition. b. The same, in the eyes-closed condition. c. epoch-SD of the log of alpha power as a function of the epoch-average log-transformed alpha power in the eyes-open condition. d. the same, in the eyes-closed condition.

*
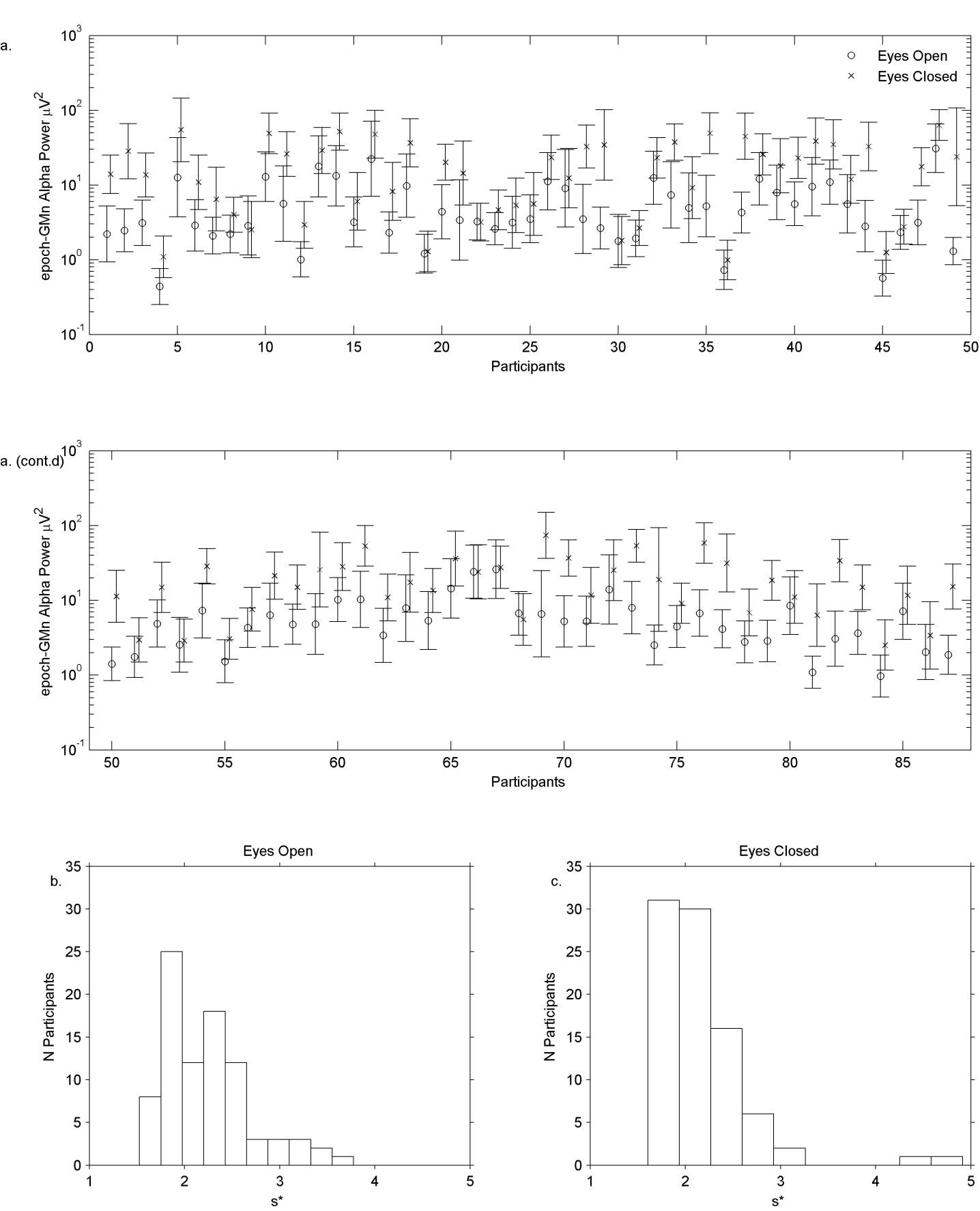
*

*Figure S4*. a. Geometric mean alpha power in the generic alpha band for all 87 participants, plotted on a log scale. Error bars indicate the multiplicative standard deviation *s** (see text). For clarity, the data for the eyes-closed condition have been slightly moved to the right. b. The distribution of the multiplicative standard deviation *s** among participants in the eyes-open condition, c. The same as (b), but in the eyes-closed condition.
