# Supplementary figures and images for: Single‐trial log transformation is optimal in frequency analysis of resting EEG alpha
Source: Eur J Neurosci. 2018 Feb 19;48(7):2585–98. doi: 10.1111/ejn.13854 (PMC6221126; doi:10.1111/ejn.13854)

EO vs. EC, GAB, all 87 participants

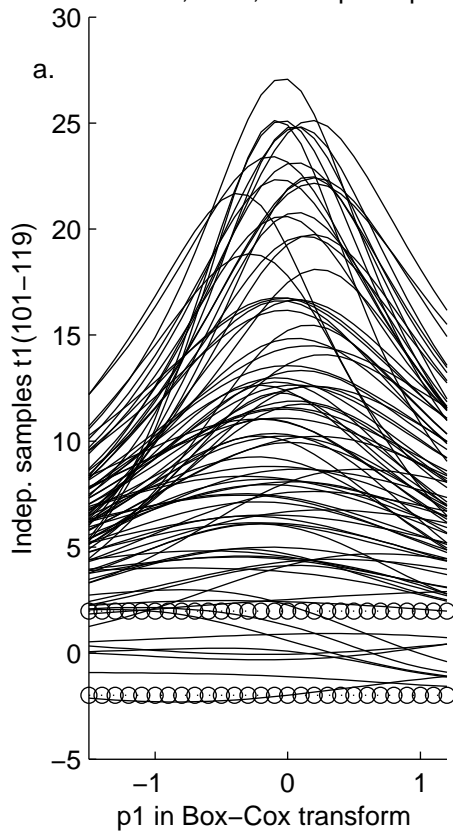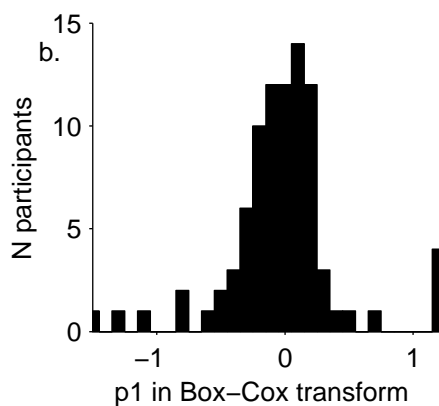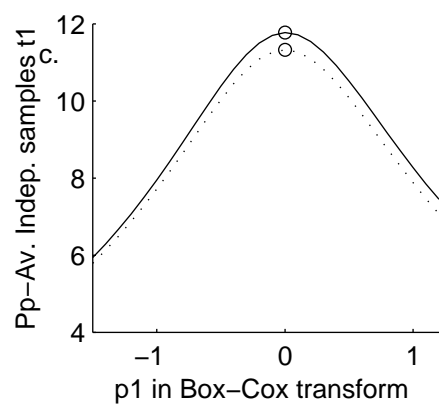

EO, GAB, all 87 participants

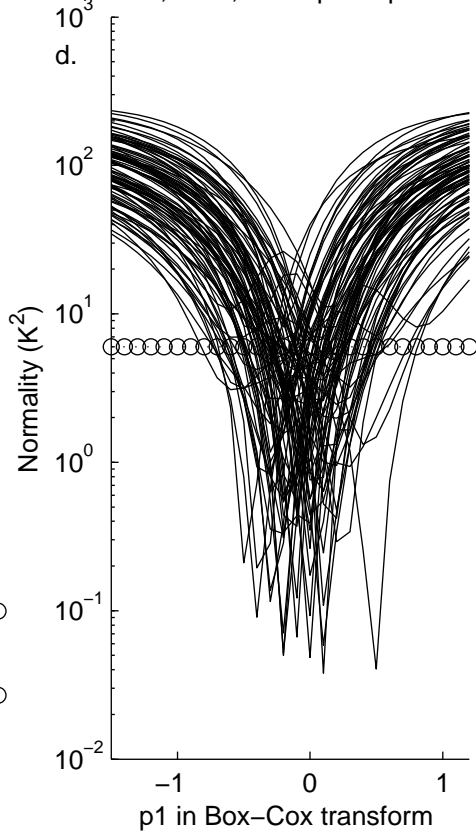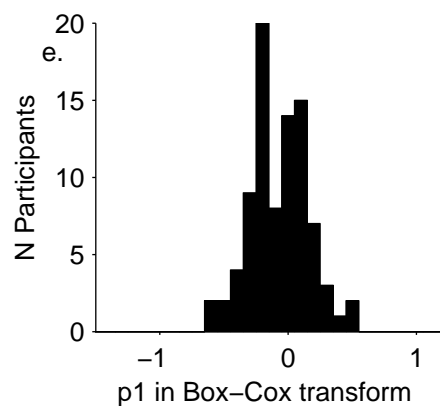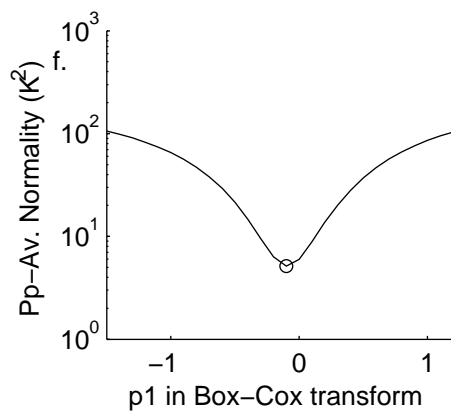

EC, GAB, all 87 participants

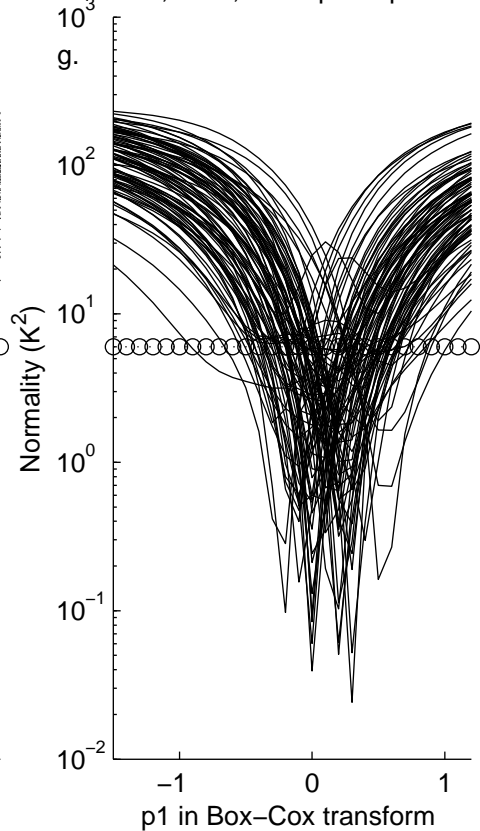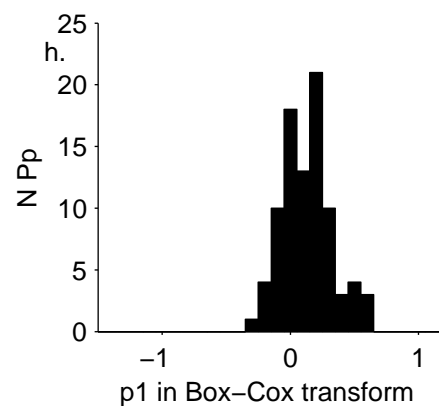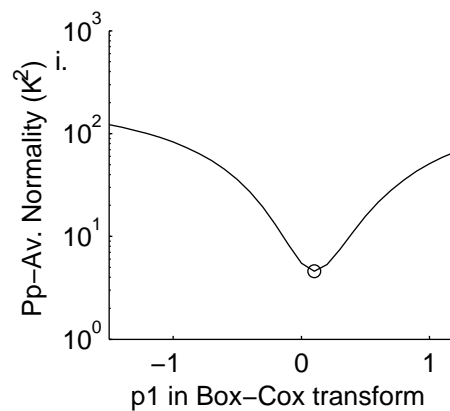

Supplement: Supplementary file 3 — Fig. S1. Effects of p1 in transformations of power in the generic alpha band at the single‐epoch level (Eq. 2) on analyses at participant level (level 1, see text). [file EJN-48-2585-s003.pdf]

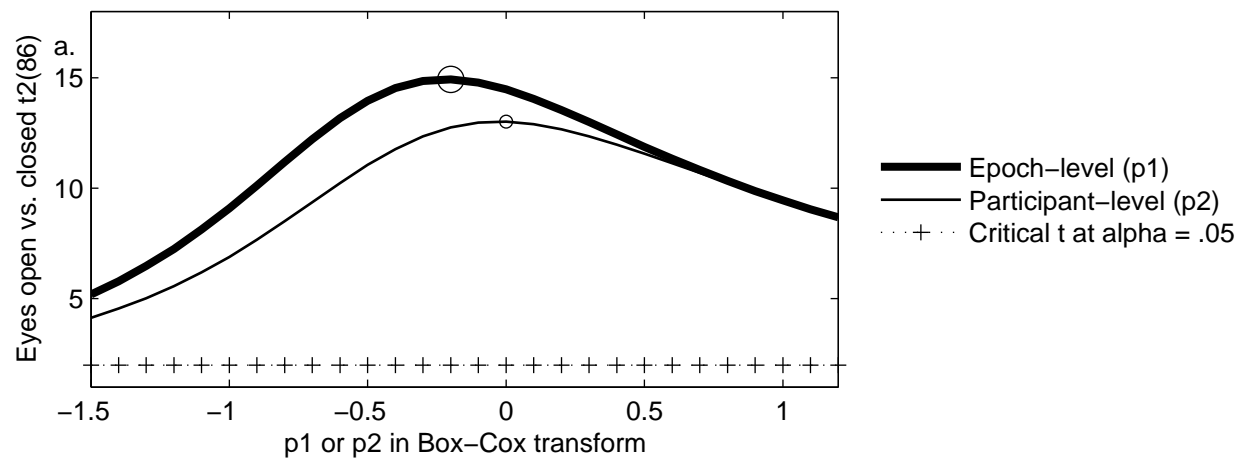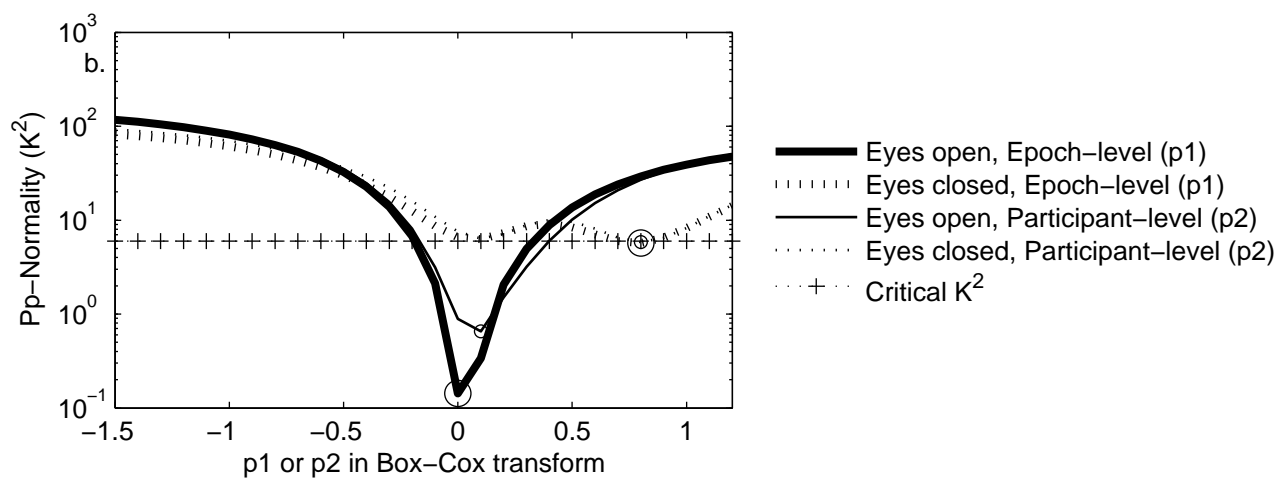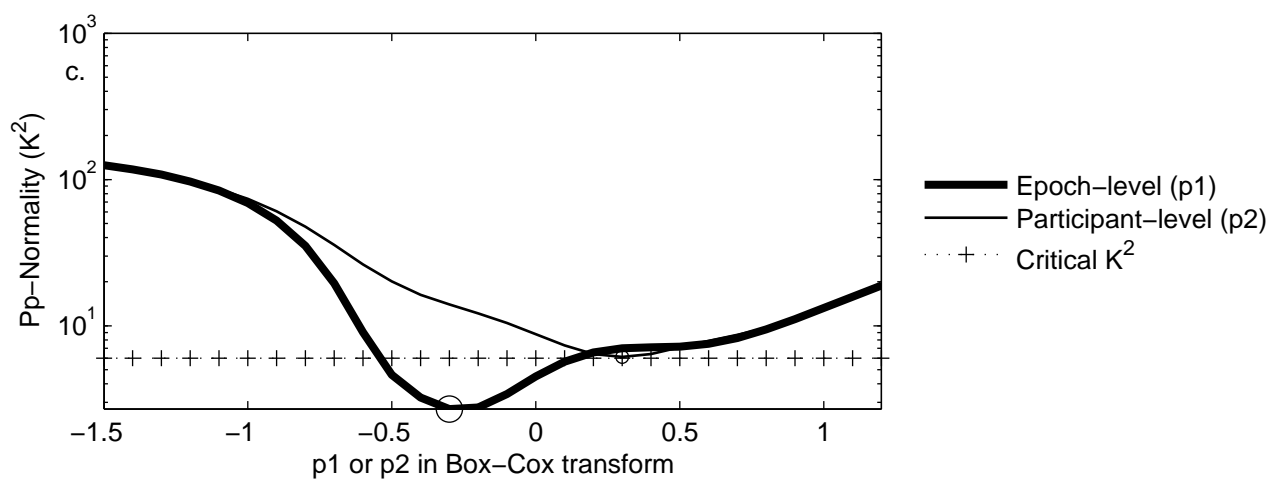

Supplement: Supplementary file 4 — Fig. S2. Effects of p1 and p2 in power transformations of power in the generic alpha band at the single epoch level (p1, Eq. 2) and epoch‐average level (p2, Eq. 4) on analyses at the group level (level 2, see text). [file EJN-48-2585-s004.pdf]

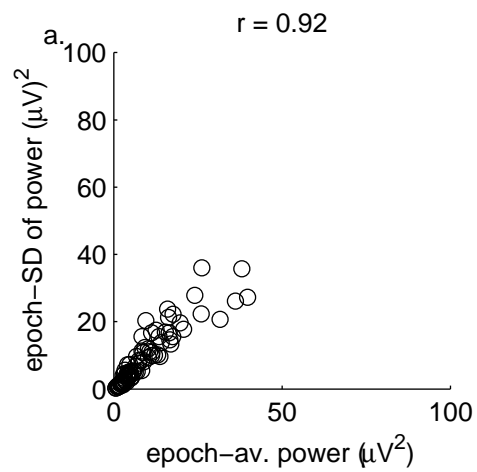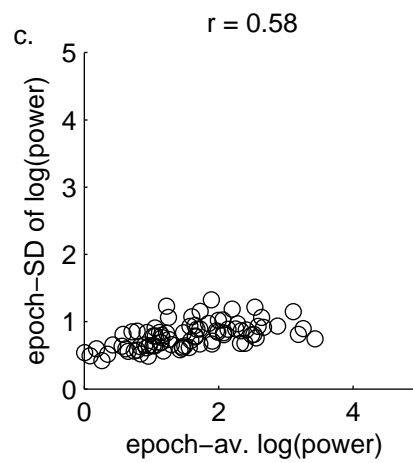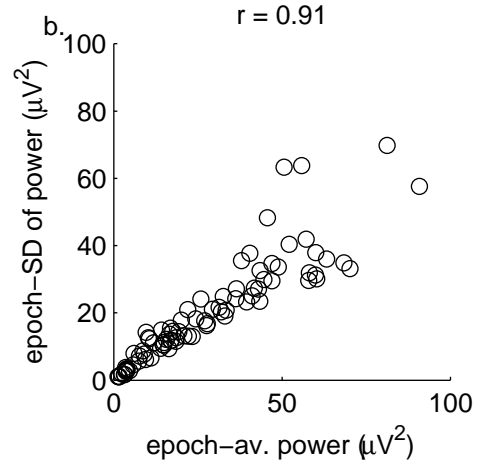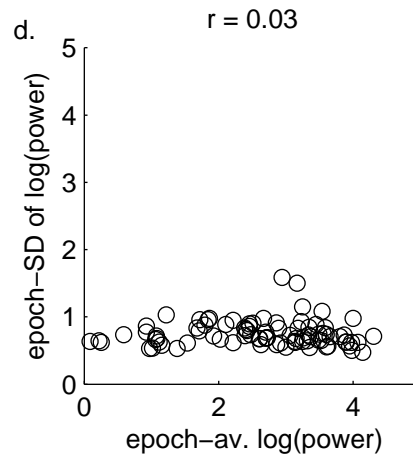

Supplement: Supplementary file 5 — Fig. S3. The relation between the average and SD of alpha magnitude across epochs in de generic alpha band. [file EJN-48-2585-s005.pdf]

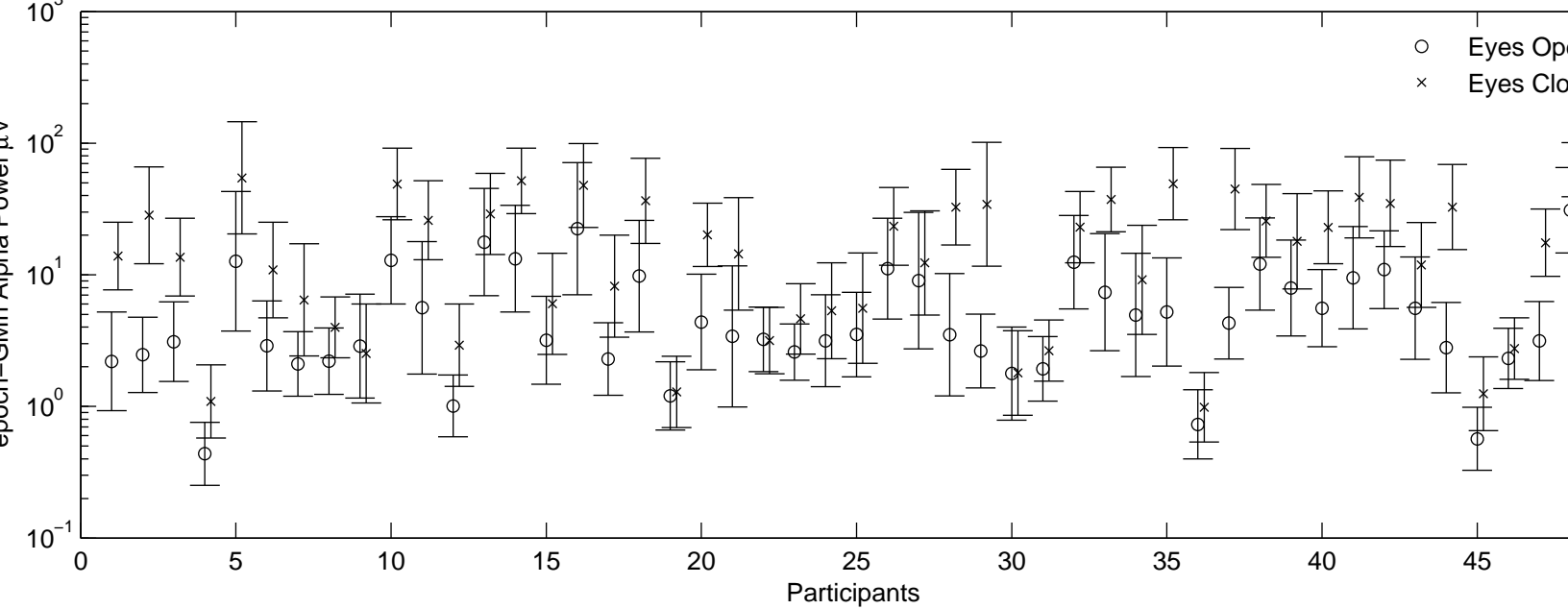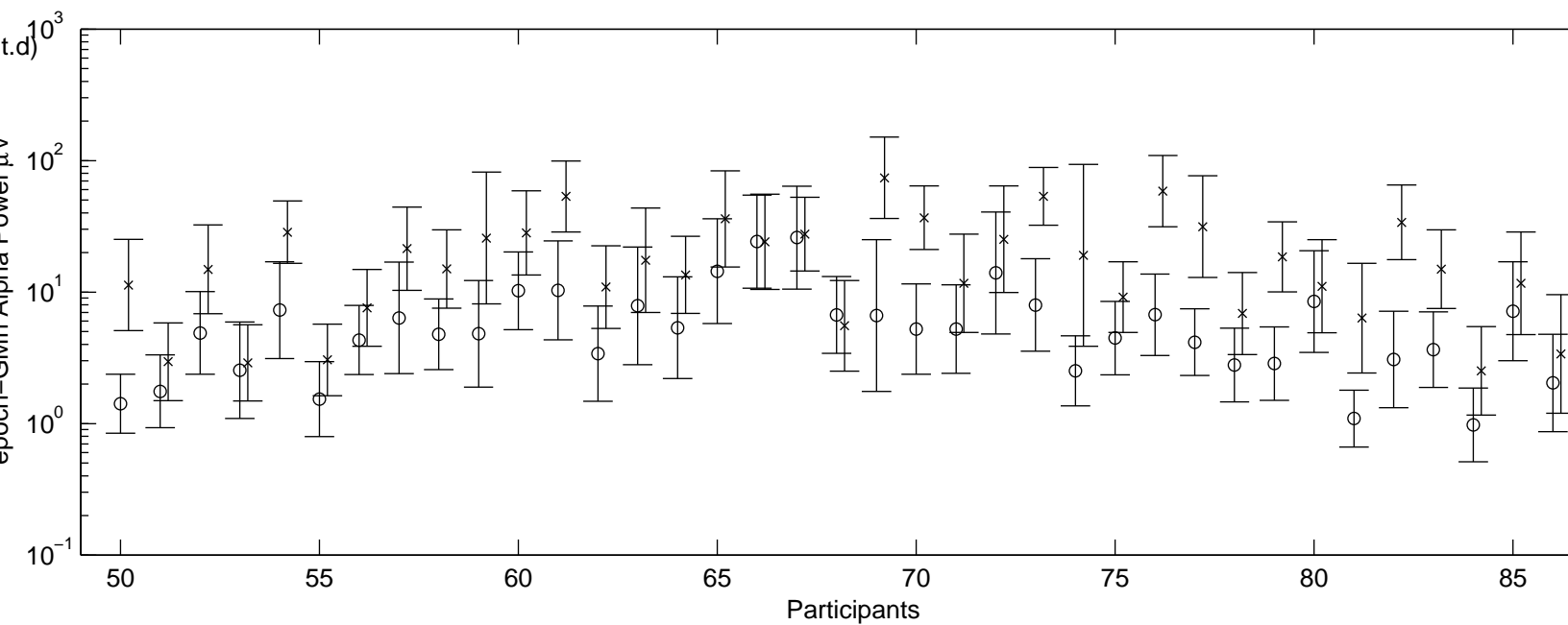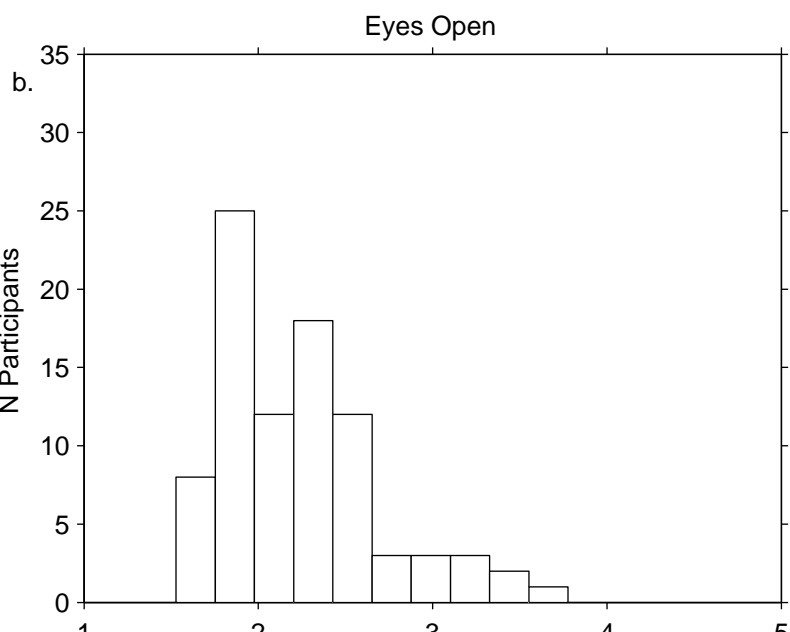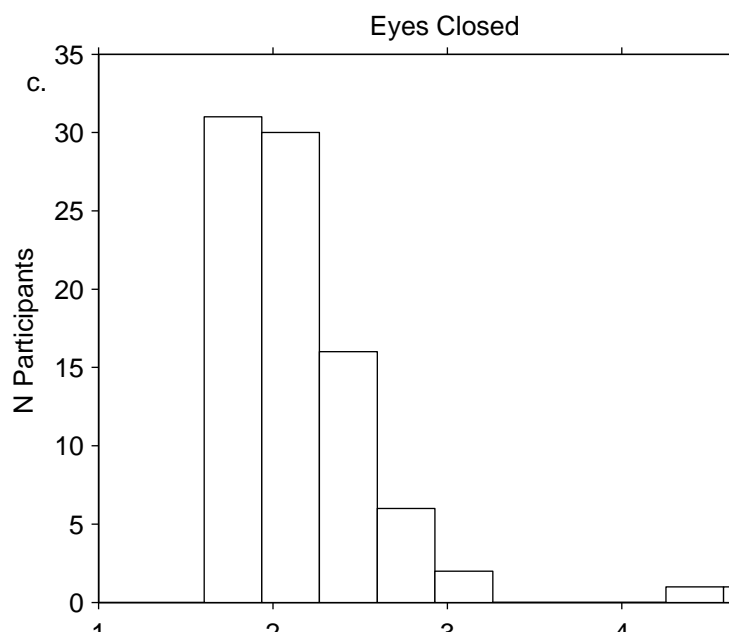

Supplement: Supplementary file 6 — Fig. S4. Geometric mean alpha power in the generic alpha band for all 87 participants, plotted on a log scale. [file EJN-48-2585-s006.pdf]
