# Supplementary material for: Single‐trial log transformation is optimal in frequency analysis of resting EEG alpha
Source: Eur J Neurosci. 2018 Feb 19;48(7):2585–98. doi: 10.1111/ejn.13854 (PMC6221126; doi:10.1111/ejn.13854)
Supplement: Supplementary file 1 [file EJN-48-2585-s001.pdf]

EJN European Journal of Neuroscience FENS Federation of European Neuroscience Societies

Single-trial log transformation is optimal in frequency analysis of resting EEG alpha

Fren T.Y. Smulders, Sanne ten Oever, Franc C.L. Donkers, Conny W.E.M. Quaedflieg & Vincent van de Ven

Review timeline:

Submission date:	27 July 2017
Editorial Decision:	06 September 2017
Revision received:	30 November 2017
Editorial Decision:	08 January 2018
Revision received:	25 January 2018
Accepted:	25 January 2018

Editor: Gregor Thut

1st Editorial Decision 06 September 2017

Dear Dr. Smulders,

Your manuscript has now been seen by two expert reviewers and one of our senior Section Editors. Based on their comments, we regret that we cannot publish your manuscript in EJN in its current form.

As you will see, both reviewers have several positive points (on questions addressed, clarity of writing, documentation of results) but also raise major concerns. The latter include low utility of the final recommendation (best transforms are already common practice, reviewer 1) and unclear generalizability to other conditions (reviewer 2), concerns about the use of not well motivated, non-exhaustive alternative transforms for comparisons (both reviewers), absence of statistics for substantiating the advantage of using IAF and other unsubstantiated claims (reviewer 2), alongside more moderate to minor points.

Addressing these concerns will likely require substantial revisions including additional analysis (addition of more standard transforms and other changes in analysis choices). In addition, we would need to be convinced of the utility and generalizability of your final recommendation. If you think you can address all points raised by the reviewers and are prepared to undertake these revisions, we invite you to resubmit a revised version.

We also noted the following points that need to be addressed in a revised version.
- Referencing is not in EJN style and the year of publication is missing for some references
- Footnotes should be removed or incorporated into the main text.
- Please carefully proof-read for the English.
- Please include the participant demographics.
- Results and discussion need to be separated out into two distinct sections.

Thank you for submitting your work to EJN and support of this Special Issue.

Kind regards,

Paul Bolam & John Foxe
co-Editors in Chief, EJN

Reviews:

Reviewer: 1 (Frederik Van Ede, University of Oxford, UK)

Comments to the Author
Review EJN-2017-07-24828(NOSC)
Smulders et al. set-out to find the optimal scaling of EEG alpha oscillations that maximised the statistical significance of the eyes-open/eyes-closed contrasts and that best approximated normality of the alpha power distributions across epochs and participants. They considered a family of non-linear transforms

(referred to as Box-Cox transforms), of which the log transform is also part. Results reveal that the optimal scaling is achieved by the log transform applied to epoch-level data.

The article is well written and the results are well documented and easy to follow. Below I elaborate on my main concerns followed by few minor suggestions.

Main
*Perhaps the main limitation is the rather limited utility of the final recommendation (the use of the log transform), provided that the log transform is already commonly used in EEG and MEG analysis of oscillatory power. Still, it is of course reassuring to see that this article's recommendation converges on common practice. Provided this quantification has not been made in this detail before (I must admit I am not sufficiently familiar with the literature on EEG scaling to properly judge this), the current work makes a relevant contribution.

*Provided there are more ways to normalise your data than by applying one of the Box-Cox transforms evaluated in this paper, it would be informative to also compare the winning Box-Cox transform (the log of the power) to these alternatives (e.g., with regard to the t-valued difference between eyes open and closed). In particular, I am considering the following alternatives (there are probably more):
-Also use amplitude instead of power, as well as log-amplitude
-Normalise the epoch-level data in the time domain (e.g., using z-scoring) before applying the FFT
-Normalise the epoch and/or participant-specific spectra as a proportion of power relative to the total power across all frequencies
-Evaluate the difference between conditions (of untransformed power or amplitude) using relative contrasts such as: [closed / open] or [(closed - open) / (closed + open)]

In other words, is the absolute difference [close - open] of the log-transformed data really the best measure?

Minor
*For me it would help if the rationale for using the Box-Cox family was better introduced
*The section on the multiplicative standard deviation was somewhat hard to parse and could benefit from providing more detail, for example in the Methods section or in the Discussion.
*The second paragraph in the discussion (support for individual alpha frequency) does not appear key to the paper (and makes a rather obvious point that individual peak frequency is not identical in different participants) and could potentially be removed to get straight to the point regarding optimal scaling. In this light, the authors may also consider revising their title.
*It would be informative also to show in the same graph the t-values when using the generic alpha and when using the individual alpha band.

Reviewer: 2 (Saskia Haegens, Columbia University, USA)

Comments to the Author

The authors test different ways of normalizing EEG power spectra, in order to maximize estimation of alpha power modulation in eyes closed vs. eyes open recordings. They also compare individual alpha peak frequency detection vs using a generic alpha band (although see my comment #3 below). The authors conclude that best results are obtained with individual bands, and log transform on the single epoch spectral level. While I find the question addressed here interesting, I have several concerns regarding the chosen approach. Furthermore, the authors make several claims that are in my opinion not substantiated by the presented data.

1) My main concern is regarding the approach used. The authors conclude: "The current results suggest that the log transform on single epochs, so preceding averaging across epochs or trials, is both necessary and sufficient. It is necessary because it converts the log-normal distribution to normal or near-normal, and transforms multiplicative effects into additive effects. It is sufficient, because it normalizes both the distribution across trials within participants, and the distribution across participants."

While I think that this main conclusion is quite sensible and good advice, I don't really understand the logic of the approach the authors use to establish this.They compare different normalization procedures, by comparing t-tests after Box Cox transformations (a method to normalize data) of the data using a range of parameters. I was not previously familiar with this method, and I am not aware of it being commonly used to normalize EEG data? That of course by itself doesn't disqualify it from being a useful procedure, but considering the outcome (basically log transform gives the best results), I am not convinced that the alternative normalizations were particularly helpful or stringent tests. I appreciate the meticulous comparison, but I don't really see the value of the range of parameters tested. Some more background and rationale for using the Box Cox transformation (and the parameters tested) would be helpful.

2) On the same topic, the authors state: “In our search for the optimal transformation, we tried many that varied in a graded way.”
I guess from the outset of the paper I expected the authors to test a range of methods that are actually commonly used in the field, and then tell us which would be the most advisable. In that sense, log transform and correcting on single epoch vs average level are good tests, and I would have expected to see these compared with other commonly used procedures, e.g., baseline correction, relative vs absolute contrasts, normalization with mean power (across spectrum), z-scoring, 1/f correction, etc. Maybe I am missing the point, but would that not be much more informative to the users of such methods? Another aspect that might be quite critical but left unexamined by the authors, is the type of referencing used for EEG acquisition (and/or subsequent re-referencing of the data).

3) Maybe I missed it, but I did not see an explicit comparison on using IAF vs the generic alpha frequency? The authors give a qualitative description of the differences, but I did not find any quantification or statistics on the advantages of using IAF here, which would actually have been very informative.

4) “Individual alpha peak frequency can be determined most reliably in only two minutes in an eyes-closed condition”
However, this is not really tested. It would have been helpful if the authors had compared IAF estimates on different subsets of the recordings, to establish how much data is required to do this (e.g., is 30 sec enough? is 5 minutes better??), and how stable these measures are over time (within a recording session).

5) If the authors want to closely examine individual peak frequencies, perhaps they should use a frequency resolution that is more fine-grained than the (somewhat oddly chosen) 0.488 Hz. Using longer segments (or zero-padding the data) would do the trick. Especially considering that peak frequency might shift between conditions, the authors might be missing that with the current suboptimal resolution.

6) “Interestingly, in many cases IAF-eceo was very close to IAF-ec.”
How is that a surprise? Power is much stronger in eyes closed than eyes open, so surely if you subtract the two the difference is going to be biased toward the condition with strongest power?

7) It would be interesting if the authors could comment as to whether applying a 1/f correction would improve the peak detection (and/or lead to different outcomes). This could be especially relevant for the eyes-open case, and/or the difference spectra, in which peaks are less pronounced than the eyes-closed case.

8) Then I have some concerns regarding the interpretation. First of all, I do not really understand the claim that “The results lent support to the concept of individual alpha frequency”. Was this up for debate? This topic has been extensively documented, going back several decades. Recently it was shown that IAF may also shift between cognitive modes (see Haegens et al Neuroimage 2014, the authors might want to cite that paper as well). Also, in context of a functional interpretation (see also my next comment) the authors might want to look into the literature on this, e.g. recent work by Samaha et al.
Second, it is not entirely clear to me what we learn regarding IAF from this study that we didn’t already know? I’m all for replication studies, but the existence of inter-individual differences in IAF has been shown extensively. Besides, if the authors are trying to make that case, they need to at the very least quantify their results on that topic, see my comments #3 and 4.

9) “Moreover IAF in the eyes closed condition is almost always close to the frequency that changes the most when the eyes are open vs. closed, and often also close to the IAF in the eyes-open condition. This can be taken as supporting evidence for a functional interpretation of individual alpha,” (page 12)
I do not understand what about this observation provides “supporting evidence for a functional interpretation”?

10) “We aimed to gather additional support for the concept of individual alpha frequency by close examination of individual EEG spectra. After that, the effect of transforms were investigated for both generic and individually defined alpha frequencies.”
The authors aim to maximize the result of a t-test eyes-open vs eyes-closed, by trying different normalizations/transformations of their data. I’m not sure why non-parametric tests were a priori excluded—these are actually a standard in the field (and for good reasons). Besides that, it is not clear to me how generalizable the current results are. Ok, now you have a procedure that maximizes the difference in posterior alpha between two baseline conditions. How do we know that result transfers to say a complex cognitive paradigm with weak expected effect size and much lower SNR? Would the authors still recommend the same procedure?

Further minor comments:

11) What is meant with "subtracting the time-mean"? (page 7, description of preprocessing)
Do the authors mean they subtracted the average voltage over time as computed per segment, or over all segments?

12) Note on figures: please put the panel labels (a,b,c, etc) outside the panels, to avoid confusion.

Authors' Response 30 November 2017

Dear Editor,
We thank you and the reviewers for your thoughtful comments. Below, we reply to each of them, and specify which changes we have made to the manuscript. We have numbered the comments from you (E1, E2, …), Reviewer 1 (R1.1, R1.2, …) and Reviewer 2 (R2.1, R2.2,…), and coded our replies as REPLY-E1, REPLY-R1.1 etc.
In the ms., textual additions and changes have been indicated by underlining.
In brief, as requested, the rationale for the Box-Cox transformations and their relation to previously described methods are better described, statistical analyses have been added, explanations have been elaborated, the title has changed, and formatting is now EJN-style.

The suggestion (reviewer 2) to use zero-paddeing before FFT has prompted us to process all data again. During the processing, we detected a participant that made some eye-blinks during the 'eyes-closed' condition, and infered that he did not consistently keep his eyes closed . We then scrutinized all raw data from our 89 participants and found one other participant with the same problem. We removed both from the analyses, so that now, n = 87. No results changed significantly. We apologize for this.
We believe the manuscript has benefitted from the changes made.

EDITOR1 (E1) low utility of the final recommendation (best transforms are already common practice, reviewer 1)
REPLY-E1: Applying the log transform on the average across epochs may be common practice, but applying it at the single epoch level, our final recommendation, is certainly not. To stress this, we changed the title to *'Single-trial log transformation is optimal in frequency analysis of resting EEG alpha'*, and also changed the abstract.

E2) unclear generalizability to other conditions (reviewer 2),
REPLY-E2. The best way to accommodate this would probably be to collect additional data, but that would add substantially to the length of the manuscript. Instead, we added a paragraph to the Limitations on p. 25 to acknowledge the limitation:

'Although measurement of resting EEG with eyes-open and eyes-closed is very common, EEG during other conditions and tasks is important in many other studies. We hypothesize that a log-normal distribution across epochs and multiplicative rather than additive effects characterize EEG in those studies as well, but this needs to be verified.'
Also, we encourage generalization in the final paragraph (p25, 26).

E3) the use of not well motivated, non-exhaustive alternative transforms for comparisons (both reviewers),
REPLY-E3. It may not be immediately obvious that the Box-Cox family of power transforms includes many that are mathematically equivalent to very common transform: square root (p = 0.5), 1/x (p = -1), cubic root (p = 1/3), untransformed power (p = 1), and the log transform (p = 0). Also, applying one member of the family of Box-Cox transform to amplitude is mathematically equivalent to applying another one to power, because (a^b)^p = a^(b*p), so there is no need to enter both amplitude (b=0.5) and power. We now explain this in the manuscript on p. 4 and p. 9, 10.
One notable exception is the relative power (power in a frequency band relative to power in all bands). Therefore, we added the latter to the analysis on p. 17, 18. It performed worse than the transformation we recommend.

EJN European Journal of Neuroscience FENS Federation of European Neuroscience Societies

E4) absence of statistics for substantiating the advantage of using IAF and other unsubstantiated claims (reviewer 2),

REPLY-E4. We added statistics for IAF vs. GAF (p. 17), and other contrasts. In some cases, however, we had difficulty finding an appropriate test (e.g. comparison of two t-values when the underlying variables are different transforms, thus not on the same scale). Fortunately the optimum (in terms of maximized t, or minimized K2) will not depend on whether mutual differences are actually statistically significant or not.

E5) We also noted the following points that need to be addressed in a revised version.

- Referencing is not in EJN style and the year of publication is missing for some references

REPLY-E5 This has been done.

- Footnotes should be removed or incorporated into the main text.

REPLY-E6. This has been done.

- Please carefully proof-read for the English.

REPLY-E7

We proof-read, and had a native speaker scrutinize the text.

- Please include the participant demographics.

REPLY-E8: These are available only for the whole population of students that were enrolled in the practical. They were assigned to small groups of at most 5, for EEG measurement in the lab. One from each group volunteered to be participant for the EEG measurement. This is now explained, and population demographics are reported on p. 6.

- Results and discussion need to be separated out into two distinct sections.

REPLY-E9. This has been done.

Reviewer: 1

R1.1) Perhaps the main limitation is the rather limited utility of the final recommendation (the use of the log transform), provided that the log transform is already commonly used in EEG and MEG analysis of oscillatory power. Still, it is of course reassuring to see that this article’s recommendation converges on common practice. Provided this quantification has not been made in this detail before (I must admit I am not sufficiently familiar with the literature on EEG scaling to properly judge this), the current work makes a relevant contribution.

REPLY-R1.1: We share with the reviewer the delight that the log transform came out as 'best', indeed exactly because it is familiar. So, what's new? First, never has it been shown to be superior among so many (28) competitors. The second innovation is to compare transforms on both the single trial level as well as on the trial-average level. The single-trial log transformation came out as best. Performed on single-trials it is certainly not common. We changed the title to stress this. See also REPLY-E1.

We added a paragraph to the introduction on p.4.

R1.2) Provided there are more ways to normalise your data than by applying one of the Box-Cox transforms evaluated in this paper, it would be informative to also compare the winning Box-Cox transform (the log of the power) to these alternatives (e.g., with regard to the t-valued difference between eyes open and closed). In particular, I am considering the following alternatives (there are probably more):

-Also use amplitude instead of power, as well as log-amplitude

REPLY-R1.2: We fully understand the concern, and we have thought of this. Mathematically, amplitude is the square-root of power. Therefore, the Box-Cox transform with p1-value of 0.5 (square root) is exactly the same as entering the amplitude, and use a p1 of 1. Therefore, 'amplitude' is already a member of the Box-Cox family (for p = 0.5).

In the same vein, log-amplitude is also included, already. It is log(power^0.5) = 0.5 * log(power), so it is a linear transform of log(power), again already a member of the Box-Cox family. The tests are not sensitive to any linear transform, so for Log-amplitude, simply look at p = 0. See also REPLY-E3

EJN European Journal of Neuroscience FENS Federation of European Neuroscience Societies

On p. 9, 10, the explanation of the Box-Cox family has been much extended.

R1.3) Normalise the epoch-level data in the time domain (e.g., using z-scoring) before applying the FFT
REPLY-R1.3: This is equivalent to using the relative spectrum. After z-scoring, SD=1, so the variance is also 1. By Parseval's theorem, the variance in the time-domain equals the sum of powers in all frequencies after FFT. Therefore, setting the variance to 1 by the zscore-transform amounts to the same as using the relative spectrum. See also REPLY-R1.4.

R1.4) Normalise the epoch and/or participant-specific spectra as a proportion of power relative to the total power across all frequencies
REPLY-R1.4: This is the relative spectrum, we now added that on p. 17, 18.

R1.5) Evaluate the difference between conditions (of untransformed power or amplitude) using relative contrasts such as: [closed / open] or [(closed - open) / (closed + open)]
REPLY-R1.5: these transforms require paired observations, thus cannot be done at single-epoch level. This is a disadvantage, because correct transformation best precedes the initial averaging across single epochs. For comparison at the average-epoch level, they would be possible, but are unlikely to perform any better (or worse) than the log transform, because they share the same feature of testing for multiplicative rather than additive effects (recall that log(a/b) = log(a) - log(b)).

Minor
R1.6)For me it would help if the rationale for using the Box-Cox family was better introduced
REPLY-R1.6 We now do this on p. 4 and p. 9, 10.
R1.7) The section on the multiplicative standard deviation was somewhat hard to parse and could benefit from providing more detail, for example in the Methods section or in the Discussion.
REPLY-R1.7 We now added better explanation on p. 18, 19.
R1.8) The second paragraph in the discussion (support for individual alpha frequency) does not appear key to the paper (and makes a rather obvious point that individual peak frequency is not identical in different participants) and could potentially be removed to get straight to the point regarding optimal scaling. In this light, the authors may also consider revising their title.
REPLY-R1.8 We completely agree that the use of Individual peak frequency has been advocated before, but felt it was worthwhile to provide additional support. For instance, our data suggest that it does not result from mere noise in the spectrum (see smooth single-peaked spectrum in the eyes-closed condition in almost all participants in Figure 2). Also, we obtained a very close correspondence between alpha peak-frequency and the frequency that displays the largest difference between eyes-open and eyes-closed spectra. Obtained at the single-participant level, the latter is not completely trivial, as one could easily imagine otherwise. See also REPLY R2.6
We revised the title in accordance with the reviewer: *'Single-trial log transformation is optimal in frequency analysis of resting EEG alpha'*

R1.9) It would be informative also to show in the same graph the t-values when using the generic alpha and when using the individual alpha band.
REPLY-R1.9: We now did that for the average t-value across participants, see Figure 4, panel c. In Figure 4, panel a, it would get lost.
Also, we now added a paragraph lending direct statistical support to the higher sensitivity of individual as opposed to generic alpha frequency on p. 17, and added this to the discussion on p. 21.

Reviewer: 2

R2.1) My main concern is regarding the approach used. The authors conclude: “The current results suggest that the log transform on single epochs, so preceding averaging across epochs or trials, is both necessary and sufficient. It is necessary because it converts the log-normal distribution to normal or near-normal, and

transforms multiplicative effects into additive effects. It is sufficient, because it normalizes both the distribution across trials within participants, and the distribution across participants."

While I think that this main conclusion is quite sensible and good advice, I don't really understand the logic of the approach the authors use to establish this.They compare different normalization procedures, by comparing t-tests after Box Cox transformations (a method to normalize data) of the data using a range of parameters. I was not previously familiar with this method, and I am not aware of it being commonly used to normalize EEG data? That of course by itself doesn't disqualify it from being a useful procedure, but considering the outcome (basically log transform gives the best results), I am not convinced that the alternative normalizations were particularly helpful or stringent tests. I appreciate the meticulous comparison, but I don't really see the value of the range of parameters tested. Some more background and rationale for using the Box Cox transformation (and the parameters tested) would be helpful.
REPLY-R2.1: The Box-Cox family includes many common transforms on EEG, but offers much more precision. We both show (Figure 1) and explain (p. 4 and p. 9, 10) that power, amplitude, 1/x, and log are actually only a few members of a larger set of transformations ('the'family') that vary in a graded way. As far as we know, testing as many as 28 members of this family, including 'conventional' power, amplitude, 1/x, and log, has not been performed before on single-epoch or average-epoch EEG. We did both. Further, the reviewer asks about stringency of the test for normality and range of parameters. Below, we address both issues.

Stringency: Indeed, the t-value associated with the t-test of the eyes-open vs. closed effect was only used to find the transform that maximizes SENSITIVITY to a widely known difference, irrespective of normality. But one possible explanation of high sensitivity could be 'close approach to normality'. To evaluate that, d'Agostino's K2 was added as the best measure of NORMALITY (Zar, 1999). Fortunately, both measures converged on pointing to the log as the best transform. We feel that introducing yet other measures of normality introduces the risk of arbitrariness. As an aside, we also tried various measures of skewness in addition to K2. They gave essentially the same results.

Range of parameters: the range was chosen to include the transforms corresponding to the common ones 'power' (p=1),' amplitude'(p=0.5), 'log'(p=0), and '1/x' (p=-1), and to extend to p=-1.5 on one side and p=1.2 on the other. All panels in Figure 4 suggest that this range was well chosen: there is only one peak, well in the center of the range. Only in panel a (t-values), there are some participants that show a clear maximum sensitivity close to the edges of the range. In these rare cases, the difference between eyes-open and closed was about zero, irrespective of the transform.

R2.2) On the same topic, the authors state: "In our search for the optimal transformation, we tried many that varied in a graded way."
I guess from the outset of the paper I expected the authors to test a range of methods that are actually commonly used in the field, and then tell us which would be the most advisable. In that sense, log transform and correcting on single epoch vs average level are good tests, and I would have expected to see these compared with other commonly used procedures, e.g., baseline correction, relative vs absolute contrasts, normalization with mean power (across spectrum), z-scoring, 1/f correction, etc. Maybe I am missing the point, but would that not be much more informative to the users of such methods?
REPLY-R2.2: As stated in REPLY-R2.1 and REPLY-E3, a number of common transforms are already members of the Box-Cox family (now made more explicit on p. 4, and p. 9, 10).
Baseline correction: not possible on resting EEG, since there is no baseline.
Relative contrast: EO / EC requires paired observations, therefore impossible on single epochs.
Normalization with mean power (across spectrum) = relative spectrum. We now added that on p. 17, 18.
z-scoring: equivalent to relative spectrum (see REPLY-R1.3).
1/f correction: see REPLY-R2.7.

Another aspect that might be quite critical but left unexamined by the authors, is the type of referencing used for EEG acquisition (and/or subsequent re-referencing of the data).
REPLY: We agree that EEG researchers face more choices, some seemingly arbitrary. We cannot solve all in this paper. The type of reference is beyond the scope of the current manuscript.

R2.3) Maybe I missed it, but I did not see an explicit comparison on using IAF vs the generic alpha frequency? The authors give a qualitative description of the differences, but I did not find any quantification or statistics on the advantages of using IAF here, which would actually have been very informative.
REPLY-R2.3: We now added a paragraph lending direct statistical support to the higher sensitivity of individual as opposed to generic alpha frequency on p. 17, and added this to the discussion on p. 21.

R2.4) “Individual alpha peak frequency can be determined most reliably in only two minutes in an eyes-closed condition”
However, this is not really tested. It would have been helpful if the authors had compared IAF estimates on different subsets of the recordings, to establish how much data is required to do this (e.g., is 30 sec enough? is 5 minutes better??), and how stable these measures are over time (within a recording session).
REPLY-R2.4: We intended to say that 2 minutes of eyes-closed EEG works well.
The word 'most' was added to stress the contrast with eyes-open EEG, not with 30 sec. and 5 minutes, which might also work well. To avoid confusion, we now removed the word 'most'. Establishing the optimal duration would be interesting, but is beyond the scope of the paper.

R2.5) If the authors want to closely examine individual peak frequencies, perhaps they should use a frequency resolution that is more fine-grained than the (somewhat oddly chosen) 0.488 Hz. Using longer segments (or zero-padding the data) would do the trick. Especially considering that peak frequency might shift between conditions, the authors might be missing that with the current suboptimal resolution.
REPLY-R2.5: Using longer segments carries the danger of violation of the assumption of stationarity, formally required for Fourier analysis. Along with other authors (e.g. Tomarken et al., 1992), we do not assume that the EEG-alpha is stationary for longer than about 2 seconds. The 0.488 Hz resolution was not purposely chosen, but dictated by our hardware, that does not allow 256 Hz sampling, which would render exactly 0.5 Hz resolution when FFT-ing over 512 points (2 seconds).
We followed the reviewer's suggestion to pad the data with zeros, doubling epoch duration. Close investigation of the signal analysis literature taught us that it does not truly increase the frequency resolution, but it can make spectra appear more smooth (Oppenheim and Schafer, 2010). All plots and analyses have been redone, and adjustments (all minor) to the manuscript have been made at many places. Nowhere were the results meaningfully different, so there was no impact on our main conclusions. On p. 7, we now state that we padded with zeros.

R2.6) “Interestingly, in many cases IAF-eceo was very close to IAF-ec.”
How is that a surprise? Power is much stronger in eyes closed than eyes open, so surely if you subtract the two the difference is going to be biased toward the condition with strongest power?
REPLY-R2.6. Admittedly, this bias plays a role, and reduces the strength of the argument to some degree. However, it is not completely obvious that a strong power in one condition must surely lead to the biggest peak in the difference as we have to consider the power in the whole frequency range that could differ between the EO and EC. For example, look at participant 13, 36, 71. All three had a strong peak for EC, but the maximum difference was not at this peak. Biologically, it seems not totally impossible that an effect similar to that observed in one of these participants were systematic across participants. Thus, by speaking against this 'not totally impossible' option, the data still carry some information.
Second, note that in 46%, the peak in EO is close to the peak in the difference (now added to results). This leads to a bias that, although smaller, opposes the bias mentioned by R2.
On a more substantive note, we do not intend to present it as a surprise, but given the importance adhered to the concept of individual alpha frequency, we still consider it worthwhile to establish. We are not

aware of this, or a similar effect, having been established before, and would be grateful for a good reference and incorporate it.
We toned down the suggestion of surprise by removing the word 'interestingly', and modified the discussion on p. 20.

R2.7) It would be interesting if the authors could comment as to whether applying a 1/f correction would improve the peak detection (and/or lead to different outcomes). This could be especially relevant for the eyes-open case, and/or the difference spectra, in which peaks are less pronounced than the eyes-closed case.
REPLY-R2.7. Indeed, 1/f correction might facilitate correct peak detection in the eyes-open condition in some cases (as would adding other tweaks to our peak detection). For various reasons, though, we prefer to not include 1/f correction. 1) We are not (yet) convinced that the meaning of the '1/f character' of EEG is so clear that it should simply be removed (as suggested by the word 'correction'), such as we do with e.g. eye-blinks. 2) In a number of cases with eyes open, alpha power is so tiny that the location of the peak will still be unreliable (and biologically virtually meaningless) after correction. 3) 1/f correction invites other frequencies to influence alpha. 4) Giving them serious attention in our analyses would encompass doing them (and also the 1/f correction + relative spectra combination) both at single epoch and average-epoch level. This would massively increase the length of our results and discussion sections.

R2.8) Then I have some concerns regarding the interpretation. First of all, I do not really understand the claim that “The results lent support to the concept of individual alpha frequency”. Was this up for debate? This topic has been extensively documented, going back several decades. Recently it was shown that IAF may also shift between cognitive modes (see Haegens et al Neuroimage 2014, the authors might want to cite that paper as well). Also, in context of a functional interpretation (see also my next comment) the authors might want to look into the literature on this, e.g. recent work by Samaha et al.
See REPLY-R1.8 and REPLY R2.6. We thank the reviewer for pointing us to the articles, and cite Haegens et al. (2014), and Samaha & Postle (2015), now.

Second, it is not entirely clear to me what we learn regarding IAF from this study that we didn’t already know? I’m all for replication studies, but the existence of inter-individual differences in IAF has been shown extensively. Besides, if the authors are trying to make that case, they need to at the very least quantify their results on that topic, see my comments #3 and 4.
See REPLY R1.8, REPLY-R1.9, REPLY2.6

R2.9) “Moreover IAF in the eyes closed condition is almost always close to the frequency that changes the most when the eyes are open vs. closed, and often also close to the IAF in the eyes-open condition. This can be taken as supporting evidence for a functional interpretation of individual alpha,” (page 12) I do not understand what about this observation provides “supporting evidence for a functional interpretation”?
See REPLY -R2.6 Also, we added an explanation of 'functional' to the discussion on p. 20 and 21. It refers to the sensitivity to an alleged difference in cortical activation.

R2.10) “We aimed to gather additional support for the concept of individual alpha frequency by close examination of individual EEG spectra. After that, the effect of transforms were investigated for both generic and individually defined alpha frequencies.”
The authors aim to maximize the result of a t-test eyes-open vs eyes-closed, by trying different normalizations/transformations of their data. I’m not sure why non-parametric tests were a priori excluded—these are actually a standard in the field (and for good reasons).
REPLY-R2.10.
Reviewing the literature, we noticed variability among articles in transformations used (amplitude, power, log, etc.), and realized that this practice has grown because magnitude of EEG-frequency has no model-dictated or 'natural' scale (as does, e.g. RT in Sternberg's additive factor method). Therefore, we considered ourselves 'free' to establish the optimal scale, using a family of carefully graded transforms. Just as others

have done on a smaller scale (Davidson et al., 1990; Gasser et al., 1982). Since non-parametric tests are insensitive to monotonic transforms, we reasoned that it makes no sense to use them to find the optimal transform.

Second, we reasoned that if we can normalize the distribution in some way, a parametric test should be more powerful than a non-parametric test.

Third, if we can linearize the measurement scale by a proper transformation, the result of any ANOVA test (additivity or interactions) would not be arbitrary as they are if data are interpreted on an ordinal scale.

R2.11) Besides that, it is not clear to me how generalizable the current results are. Ok, now you have a procedure that maximizes the difference in posterior alpha between two baseline conditions. How do we know that result transfers to say a complex cognitive paradigm with weak expected effect size and much lower SNR? Would the authors still recommend the same procedure?

REPLY-R2.11. If it is accepted that our data suggest that effects on alpha magnitude are multiplicative in nature (normality after log-transform suggests log-normality of untransformed distribution, suggests multiplicative character; SD increases linearly with mean), then it seems plausible that also smaller effects are multiplicative. But, indeed that must be verified.

We added a paragraph to the Limitations on p. 25 and our final recommendation on p. 25, 26. See also REPLY-E2

Further minor comments:

R2.12) What is meant with “subtracting the time-mean”? (page 7, description of preprocessing) Do the authors mean they subtracted the average voltage over time as computed per segment, or over all segments?

REPLY-R12 We subtracted the mean per segment (removing the 0 Hz component), which makes sense if one tapers with a window. We now removed the word 'whole' to avoid the confusion.

R2.13) Note on figures: please put the panel labels (a,b,c, etc) outside the panels, to avoid confusion.

REPLY-R13 This has now been done.

2nd Editorial Decision 08 January 2018

Dear Dr. Smulders,

Your resubmitted manuscript has now been re-evaluated by the original reviewers as well as by the Section Editor, Prof. Gregor Thut, and ourselves. We are pleased to inform you that we are provisionally accepting the paper for publication in EJN pending one last minor revision, which will not require re-review.

In your final version, we ask that you to take into consideration the final comment of reviewer 2 on a limitation of the study (copied below), namely to make sure this limitation is acknowledged in the paper.

Comment of reviewer 2:
The authors adequately replied to most of my previous concerns. However, considering their reply to my previous comment 2.2, I remain that the utility of the findings is rather limited. The recommendations only hold for resting state EEG measures, not for typical task-based paradigms (since for that, they should indeed have tested all the options I listed in my original comment, and which the authors claim do not apply to resting state data).

Please also ensure that you provide a text file and a figure file for the Graphical Abstract (as detailed in the instructions below).

If you are able to respond fully to the points raised, we would be pleased to receive a revision of your

paper within 30 days.

Thank you for submitting your work to EJN.

Kind regards,

John Foxe & Paul Bolam
co-Editors in Chief, EJN

Reviews:

Reviewer: 1 (Frederik Van Ede, University of Oxford, UK)

Comments to the Author
Well reasoned and well argued revision. I have no further concerns.

Reviewer: 2 (Saskia Haegens, Columbia University, USA)

Comments to the Author
The authors adequately replied to most of my previous concerns. However, considering their reply to my previous comment 2.2, I remain that the utility of the findings is rather limited. The recommendations only hold for resting state EEG measures, not for typical task-based paradigms (since for that, they should indeed have tested all the options I listed in my original comment, and which the authors claim do not apply to resting state data).

Authors' Response 25 January 2018

Dear Editor,
We thank you and the reviewers for constructive comments and the positive evaluation. Please find below our reply to the final comment of reviewer 2, and the changes we made.
Minor other changes: we noticed that we previously accidentally uploaded the wrong version of Figure 5 (the Supplementary one, Figure S5 instead of Figure 5), and made a minor error in the caption of Supplementary Figure S1. Both changes make no significant difference. We corrected both.

Comment of reviewer 2:
The authors adequately replied to most of my previous concerns. However, considering their reply to my previous comment 2.2, I remain that the utility of the findings is rather limited. The recommendations only hold for resting state EEG measures, not for typical task-based paradigms (since for that, they should indeed have tested all the options I listed in my original comment, and which the authors claim do not apply to resting state data).

Reply:
For task-related changes, indeed the amount of potentially useful transformations may be larger than for resting EEG, and we only studied resting EEG. We explicitly acknowledge that and refer to Grandchamps and Delorme (2011) as a good reference on task-related effects.
We now added the following sentences to the Limitations paragraph (text in capitals is new).

THE CURRENT RESULTS CONCERN ONLY RESTING EEG. Although measurement of resting EEG with eyes-open and eyes-closed is very common, EEG during other conditions is important in many other studies. We hypothesize that a log-normal distribution across epochs and multiplicative rather than additive effects characterize EEG in those studies as well, but this needs to be verified. ALSO, IN TASK-BASED PARADIGMS EFFECTS MIGHT BENEFIT FROM TRANSFORMATIONS NOT TESTED HERE. BASED ON OUR RESULTS IN THE EYES-OPEN CONDITION, SUGGESTING A LOGNORMAL DISTRIBUTION ALONG SINGLE-EPOCHS EVEN WHEN ALPHA MAGNITUDE IS COMPARATIVELY SMALL (FIGURE 4D, E, F), WE WOULD EXPECT BENEFICIAL

EJN European Journal of Neuroscience FENS Federation of European Neuroscience Societies

EFFECTS ESPECIALLY FOR TRANSFORMS THAT CAPITALIZE ON PROPORTIONAL RATHER THAN ADDITIVE TASK EFFECTS (SEE GRANDCHAMPS & DELORME, 2011).
